# A hierarchy of selection pressures determines the organization of the T cell receptor repertoire

**DOI:** 10.3389/fimmu.2022.939394

**Published:** 2022-07-29

**Authors:** Michal Mark, Shlomit Reich-Zeliger, Erez Greenstein, Dan Reshef, Asaf Madi, Benny Chain, Nir Friedman

**Affiliations:** ^1^ Department of Immunology, Weizmann Institute of Science, Rehovot, Israel; ^2^ Department of Pathology, Tel-Aviv University, Tel-Aviv, Israel; ^3^ Department of Computer Science, University College London, UCL, London, United Kingdom

**Keywords:** TCR repertoire, CDR3AA motifs, LCMV, aging, epitope-specific repertoire

## Abstract

We systematically examine the receptor repertoire in T cell subsets in young, adult, and LCMV-infected mice. Somatic recombination generates diversity, resulting in the limited overlap between nucleotide sequences of different repertoires even within the same individual. However, statistical features of the repertoire, quantified by the V gene and CDR3 k-mer frequency distributions, are highly conserved. A hierarchy of immunological processes drives the evolution of this structure. Intra-thymic divergence of CD4+ and CD8+ lineages imposes subtle but dominant differences observed across repertoires of all subpopulations in both young and adult mice. Differentiation from naive through memory to effector phenotype imposes an additional gradient of repertoire diversification, which is further influenced by age in a complex and lineage-dependent manner. The distinct repertoire of CD4+ regulatory T cells is more similar to naive cells in young mice and to effectors in adults. Finally, we describe divergent (naive and memory) and convergent (CD8+ effector) evolution of the repertoire following acute infection with LCMV. This study presents a quantitative framework that captures the structure of the repertoire in terms of its fundamental statistical properties and describes how this structure evolves as individual T cells differentiate, migrate and mature in response to antigen exposure.

## Introduction

The ability to sustain effective T cell immunity relies on a diverse αβ heterodimeric T cell receptor (TCR) repertoire generated by the stochastic variable, diversity and joining (VDJ) recombination mechanism (1). This diverse repertoire is shaped over time by recombination biases (2, 3), thymic and extra-thymic selection (1, 2, 4), selective migration, and antigen-driven clonal expansion. The encounter with cognate peptide-MHC complex (pMHC) also drives the differentiation of the T cell. For example, the strength of TCR stimulation can skew differentiation of memory versus effector T cells (3, 4) and CD4+ regulatory (Treg) versus effector/memory CD4+ cells (5, 6), linking TCR specificity to phenotype and function. Individual components of this complex process have, of course, been documented previously. For example, significant changes can be found between the repertoires of CD4+ and CD8+ cells, presumably reflecting selection by different classes of MHC peptide complexes (7, 8). Similarly, the repertoire differences between CD4+ Treg and conventional CD4+ cells (9, 10) are presumed to be shaped by their recognition of self or foreign peptides. The novel aim of this study is to integrate these diverse processes by comprehensively analyzing the changing structure and organization of the TCR repertoire across subsets, tissues, and ages, creating a high-level view of the hierarchy that governs them.

In young individuals, the majority of the T cell compartment is made up of naive cells, and the repertoire is shaped largely by stochastic recombination and thymic selection. However, as individuals age, their immune system responds to an increasing number of foreign antigens, derived principally from microbial, allergen, or altered-self (e.g., neoantigen) exposure. This drives a shift towards the memory/effector phenotype (11), accompanied by increased clonal expansion. Interestingly, exposure to antigens in different individuals can drive both convergent and divergent repertoire evolution (12, 13). At the repertoire level, clonal expansion results in a gradual decrease in overall repertoire diversity (14, 15). The Treg repertoire also changes with age, as production of thymic “natural” Treg drops significantly, and these are replaced by a high proportion of Tregs with active effector/memory phenotype (16, 17).

In this study, we combine multi-parameter fluorescence-activated cell sorting with high-throughput-next generation sequencing to undertake a comprehensive high resolution analysis of the αβ TCR repertoire of various T cell compartments in young and adult mice, comparing CD4+ and CD8+ T cells of naive, central memory, effector and Tregs, from the spleen and bone marrow. We also examine the impact of acute strong antigen exposure by analyzing the changes that follow infection with lymphocytic choriomeningitis virus (LCMV). We quantify the global parameters of the repertoire at different levels of dimensionality, spanning variable gene frequencies, linear amino acid motifs (k-mer) frequencies and at the level of individual nucleotide sequences. The underlying hypothesis that we seek to test is that despite the stochastic nature of the repertoire generation process, we can identify a clearly defined structure in the repertoire which is highly conserved between different individuals and that exposure to antigen and the environment drives changes in this structure.

We demonstrate that, although the stochasticity of the TCR generation process generates so much diversity that there is little overlap between repertoires at the level of individual TCR nucleotide sequences, statistical averaging over thousands of TCRs creates a stable structure which can be captured by statistical parameters such as V gene distributions and CDR3 amino acid k-mer frequency distribution. We use this quantitative framework to document a hierarchy of processes which drive repertoire evolution, driven by lineage development, functional differentiation, age and migration. Age and differentiation interact in a complex manner, which is unexpectedly different between CD4+ and CD8+ lineages. Paradoxically, we find that the stochastic process of thymic repertoire generation generates a highly conserved and stable structure. But subsequent exposure to antigens and the environment, even in individuals who are genetically identical and live in the same controlled environment, drive idiosyncratic changes and repertoire diversification.

## Materials and methods

### Animals

All experiments except for the LCMV infections were carried out using six inbred female *Foxp3* ^GFP^ (C57BL/6 background) mice which were sacrificed at three months (young) and one year (adults). All animals were handled according to regulations formulated by The Weizmann Institute’s Animal Care and Use Committee and maintained in a pathogen-free environment.

### LCMV infections

Seven female C57BL/6 mice at five weeks old (Envigo) were injected intraperitoneally with the Armstrong LCMV strain. Mice were collected after 8 or 40 days of infection.

### Sample preparation and T cell isolation

Spleens were dissociated with a syringe plunger, and single-cell suspensions were treated with ammonium-chloride-potassium lysis buffer to remove erythrocytes. Bone marrows were extracted from the femur and tibiae of the mice and washed with PBS. Samples were loaded on the MACS column (Miltenyi Biotec), and T cells were isolated according to the manufacturer’s protocol. Bone marrow cells were purified with CD3+ T isolated kit (CD3ϵ MicroBead Kit, mouse, 130-094-973, Miltenyi Biotec). Splenic CD4+ and CD8+ cells were purified in two steps (1): CD4+ positive selection (CD4+ T Cell Isolation Kit, mouse, 130-104-454, Miltenyi) (2) the fraction of the negative cells were further selected for the CD8+ positive cells (CD8a+ T Cell Isolation Kit, mouse, 130-104-07, Miltenyi Biotec). For the tetramers binding reaction, we pooled splenocytes from vaccinated mice (5 mice after eight days of infection) and purified their T cells using the untouched isolation kit (Pan T Cell Isolation Kit II, mouse, 130-095-130, Miltenyi Biotec).

### Flow cytometry analysis and cell sorting

The following fluorochrome-labeled mouse antibodies were used according to the manufacturers’ protocols: PB or Percp/cy5.5 anti-CD4, PB or PreCP/cy5.5 anti-CD8, PE or PE/cy7 anti-CD3, APC anti-CD62L, Fitc or PE/cy7 anti-CD44 (Biolegend). Cells were sorted on a SORP-FACS-AriaII and analysed using FACSDiva (BD Biosciences) and FlowJo (Tree Star) software. Sorted cells were centrifuged (450g for 10 minutes) prior to RNA extraction.

### LCMV tetramers staining and FACS sorting

Three monomers (NIH Tetramer Core Facility) with different LCMV epitopes were used: MHCI- NP396-404(H-2Db), MHCI- NP205-212(H-2Kb), MHCI-GP92-101 (H-2Db). Tetramers were constructed by binding Biotinylated monomers with PE/APC-conjugated-streptavidin (according to the NIH protocol). Purified T cells were stained with FITC anti-CD4 and PB anti-CD8 and followed by tetramers staining (two tetramers together) for 30 min at room temperature (0.6ug/ml). CD8+ epitope-specific cells were sorted from single-positive gates for one type of tetramer.

### Library preparation for high-throughput TCR sequences

All libraries in this work were prepared according to the published method (18), with minor adaptations for mice. Briefly, we extracted total RNA from CD4+/CD8+/CD3+ T cells (from spleen or bone marrow) of *Foxp3* ^GFP^ or C57BL/6 mice using RNeasy Micro Kit (Qiagen) and cleaned from excess DNA with DNAse 1 enzyme (Promega). RNA samples were reverse transcribed to cDNA, and an anchor sequence at the variable part of the TCR was added using single-strand ligation. Ligation products were amplified by PCR in three reactions, using an extension PCR to add Illumina sequencing primers, indices, and adaptors. Our modified protocol for mice included specific primers for the constant region of the TCR α or β chain (GAGACCGAGGATCTTTTAACTGG with GCTTTTGATGGCTCAAACAAGG, for α and β chains, respectively). These primers are used in the reverse transcription (RT) and the first two PCR reactions (PCR: CAGCAGGTTCTGGGTTCTGGATG with TGGGTGGAGTCACATTTCTCAGATCCT for α and β chains, respectively). Primers in the second round of the PCR included TCR constant region sequence, together with a six base pair Illumina index for multiplex sequencing, six random base pairs to improve cluster calling at the start of read 1, and the Illumina SP1 sequencing primer (PCR2: ACACTCTTTCCCTACACGACGCTCTTCCGATCTHNHNNH-index-CAGCAGGTTCTGGGTTCTGGATG with ACACTCTTTCCCTACACGACGCTCTTCCGATCTHNHNNH-index-GGTGGGAACACGTTTTTCAGGTCCTC for α and β chains, respectively). In the third round of the PCR, the primers were the SP1 and SP5 Illumina adaptors (PCR3: CAAGCAGAAGACGGCATACGAGAT with AATGATACGGCGACCACCGAGATCTACACTCTTTCCCTACACGACGCTCTTCC, forward and reverse, respectively). All PCR reactions were done using KAPA HiFi high-fidelity proof-reading polymerase (KAPA Biosystems). Libraries were sequenced using NextSeq 550 (200 bp forward read, 100 bp reverse) (Illumina).

### Pre-processing and error correction for raw reads

Data was processed using an in-house pipeline, coded in R. First, we transferred the UMI sequence from the read2 to read1 sequence. Trimmomatic (19) was used to filter out the raw reads containing bases with Q-value ≤20 and trim reads containing adaptors sequences. The remaining reads were separated according to their barcodes, and reads containing the constant region for α, or β chain primers sequences were filtered (CAGCAGGTTCTGGGTTCTGGATG/TGGGTGGAGTCACATTTCTCAGATCCT for α and β chains respectively), allowing up to three mismatches. Bowtie 2 (20) (using sensitive local alignment parameters) was used to align the reads to the germline V/J gene segments, as found in the IMGT. The CDR3 nucleotide sequences were translated to amino-acid sequences in two steps. The N-terminal Cysteine was identified by matching it to the V-aligned region. Then the C-terminal Phenylalanine was identified by matching it to the J-aligned region. Up to one mismatch was allowed per 18-stretch sequence, ending with the Cys or starting at the Phe. CDR3AA sequences were defined according to the IMGT convention. To correct for possible sequence errors, we cluster the sequences UMIs in two steps (1); UMIs with the highest frequency were grouped within a Levenshtein distance of 1 (2). Out of these sequences, CDR3AA sequences (starting from the most frequent sequence in a group) were clustered using a Hamming distance (21) threshold of 4. These thresholds were based on predicted sequence errors (quality scores >30) per sequence length (22, 23). Finally, the UMIs of each CDR3 sequence were counted, and UMI count reads with one copy number were filtered out. For the entire analysis, we used the fully annotated sequences (both V and J segments assigned), in-frame (i.e., encode for a functional peptide without stop codons), and copy numbers greater than one. In addition, we removed the invariant α chain of the iNKT CDR3 sequence (CVVGDRGSALGRLHF (24), 0.001% from all sequences in our data).

### Analysis

All statistical analysis was performed using R Statistical Software (version 4.0.0). For the pre-processing pipeline, we used the “ShortRead” package (version 1.48.0) (25). The package “vegan” (version 2.5-7) (26) was used to calculate the Simpson (27), Horn-Morisita indices (28, 29), and to project Nonmetric Multidimensional Scaling (30). The Cosine similarity was computed with the package “coop” (version 0.6-3) (31). The Horn-Morisita and the Cosine indices rely on both overlap and abundancy of sequences, as evaluated by the unique molecular identifier count (UMI count) (32, 33). The indices are calculated according to the following two equations:


$$\text{Horn index}=\;\frac{2x\cdot y}{∥x{∥}^{2}+∥y{∥}^{2}}=\frac{2{\sum^{}}_{i}x_{i}y_{i}}{{\sum^{}}_{i}x_{i}^{2}+{\sum^{}}_{i}y_{i}^{2}}$$

$$\text{Cosine}\left(x,y\right)=\frac{x\cdot y}{∥x∥\;∥y∥}=\frac{{\sum^{}}_{i}x_{i}y_{i}}{\sqrt{{\sum^{}}_{i}x_{i}^{2}{\sum^{}}_{i}y_{i}^{2}}}$$

The vectors x and y represent the abundance of each TCR in the two immune repertoires sequences to be compared. T cell repertoires were sub-sampled for equal size (n=1000/500 CDR3NTβs clones in spleen or bone marrow, respectively). CDR3NT sequences were replicated according to the UMI count number and then randomly sampled. The average Simpson and Shannon diversity scores were calculated from 30 repeats of this random sampling.

The Davies-Bouldin’s index (34) was calculated using the “clusterSim” package (version 0.49-2) (35) according to the equation:

$DB\;index=\frac{1}{N}\overset{i=1}{\underset{N}{\sum^{}}}\underset{j\neq i}{\max}\frac{S_{i}+S_{j}}{M_{i,j}}$ where S_i_ is the standard-deviation of all vectors belonging to cluster i

( $S_{i}=\sqrt{\frac{1}{\left|C_{i}\right|}\sum_{j\in Ci}∥x_{j}-\mu_{i}{∥}_{2}^{2}}$ and M_i,j_ is L2-distance between the cluster centroids *M_i_*, *_j_* = ||*µ_i_* – ||*µ_j_*_2_

For the PCA analysis, we applied the “factoextra” package (version 1.0.7) (36), and the “ggplot2” (version 3.3.5) (37) was used for generating figures. The sequential triplets (38) and N-terminal 7-mer motifs were extracted from the full CDR3AA sequences of each mouse, compartment, and chain. The frequency of each motif was calculated from the UMI count of the original CDR3AA sequence and normalized by compartment and age group. Since the number of all possible triplets combinations (8000) and observed heptamers (~ 46 and 165 thousand) was high, we reduced the noise by focusing on the most frequently expressed motifs. Triplets were filtered based on the mean frequency of each sequence across all compartments and mice (400 and 2500 in the β and α chain, respectively). Heptamers were selected in two subsequent steps: 1) top 25 from each sample and (2) top 150 motifs according to the mean frequency of each sequence across all compartments and mice.

Synthetic repertoires (control 1) were generated using SONIA (39) using the default model of repertoires adjusted for global features of thymic selection. We generated three artificial repertoires of the same size as the mean repertoire size of the three young mice and then randomly allocated the TCRs to different compartments.

We also generated an additional set of synthetic repertoires (control 2) by combining the unique TCR sequences (with the V and J annotations) from all CD4+ or CD8+ compartments in all mice. For each subpopulation in each young mouse, we replaced the TCR in the actual repertoire with a random TCR from the combined set while keeping the abundance the same. We, therefore, constructed controls for each subpopulation whose abundance profile was the same but whose TCR sequences were randomly assigned. The cosine scores or frequencies of these control 2 populations were computed by averaging values over 100 repeat samplings.

### Data availability

All cDNA sequences from young and adult mice have been submitted to the Sequence Read Archive under identifier PRJNA771880. https://www.ncbi.nlm.nih.gov/Traces/study/?acc=PRJNA771880&o=acc_s%3Aa

## Results

### A quantitative description of the TCR repertoire

**Figure 1:**
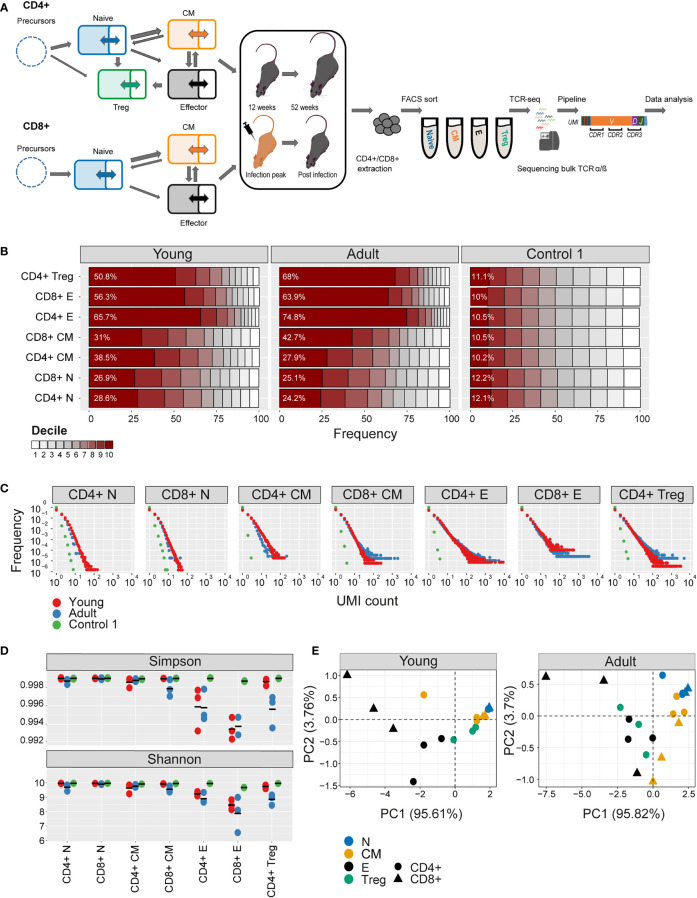
Clonal expansion and diversity of the TCRβ repertoire in different subsets of young and adult mice. **(A)** Summary of T cell compartments and pipeline for cell isolation and TCR repertoire sequencing and analysis. **(B)** The TCRs in each repertoire were ranked according to frequency, and the proportion within each decile is illustrated (low abundance sequences in white, ranging to high abundance sequences in dark red). The percentage of the distribution represented by the top decile is shown in white text. **(C)** The sequence abundance distribution in each compartment. The plots show the proportion of the repertoire (y-axis) made up of TCR sequences observed once, twice, etc. (x-axis). Repertoires from young mice are shown with red dots, repertoires from older mice in blue dots, and synthetic repertoires in green. **(D)** Simpson and Shannon scores for subsampled repertoires of equal size (1000 CDR3NTs) from each compartment and mouse. Colors same as panel **(C)** Mean is shown as black lines (n=3). **(E)** PCA of the Renyi diversities of order 0, 0.25, 0.5, 1, 2, 4.

We collected CD4+ and CD8+ T cells of naive, central memory, effector, and Tregs, from the spleen and bone marrow of 12 and 52-week-old mice (summarized in **Figure 1A**). Representative flow cytometry plots showing the phenotypic markers, the gating strategy, and relative purity of the populations obtained are shown in supplementary (**Figure 1-figure supplement 1A, B**). We appreciate that our antibody panel does not fully capture the complexity of the T cell compartment and that more extensive panels would be required to fully differentiate between all the known sub-compartments. However, for the purpose of this high-level analysis, we simplify the nomenclature and refer to the sorted populations as naive, Treg, central memory, and effector. After RNA extraction, we amplified the TCR repertoire using a previously published experimental pipeline which incorporates unique molecular identifiers (UMI) for each cDNA molecule to correct for PCR bias and sequencing error, allowing a robust and quantitative annotation of each sequence in terms of V gene, J gene, CDR3 sequence and frequency (18, 40).

The numbers of cells and the number of TCR mRNAs (captured by the total UMI count) which were recovered varied widely between compartments and age groups. For example, both splenic CD4+ and CD8+ naive compartment from young mice resulted in the highest average UMI count (~415,000), while the splenic CD4+ central memory (CM) population yielded the lowest average UMI count (~44,000). As expected, the proportion of naive cells in both spleen and bone marrow was higher in young than adult mice, and this was balanced by an increase in memory and especially effectors in the older mice (**SI Table 1**). The total UMI count was strongly correlated with the number of sorted cells across compartments and tissues (**Figure 1-figure supplement 1C**). The number of α and β UMIs were also highly correlated (**Figure 1-figure supplement 1D**). Both these correlations provide additional confidence in the robustness and quantitative output of the overall pipeline.

### The clonal structure and diversity of the repertoire vary with compartment and age

We first explored the changes in the clonality and diversity of the TCR repertoire across compartments and tissues. We estimated T cell clonotype size by the number of different UMIs associated with a unique TCR and illustrated the clonal frequency distribution of the repertoire within each population (**Figures 1B, C** for spleen; **Figure 1- figure supplement 2A, B** for bone marrow). Clonal distribution normalized for sample size (total UMI count) and cumulative frequency distributions are displayed in **Figure 1- figure supplement 3**. As a comparator in this, and subsequent figures, we generated a set of synthetic TCRs using SONIA, a generative probabilistic model of TCR recombination which incorporates learnt parameters of the genomic TCR recombination process, without any subsequent selective expansion (39). These synthetic sequences, which we refer to as control 1, serve as one baseline with which to compare real repertoires, in which the products of recombination have been shaped by selection and proliferation. Other approaches to constructing control populations are discussed below.

As expected, the naive repertoires were dominated by rare TCRs and had few expanded clonotypes (the darkest color in **Figure 1B** and the points to the right in **Figure 1C**; see also **Figure 1- figure supplement 3**). The naive repertoires were also most similar to the synthetic repertoires. In contrast, T effectors contained much larger numbers of expanded clonotypes, especially in CD8+ cells from the older mice. The Simpson and the Shannon indices, two commonly used measures of repertoire diversity, were highest in naive populations from young individuals, and progressively lower in central memory and effectors (**Figure 1D**, **Figure 1- figure supplement 2C**; note that there were insufficient naive cells from bone marrow to generate TCR libraries). The Simpson and Shannon indices are examples (k = 2 and k = 1, respectively) of a series of diversity measurements, which are captured by the Renyi entropy of order k, where k can run from 0 to infinity. We calculated the Renyi diversities for k = 0, 0.25, 0.5, 1, 2, 4 for each repertoire and then plotted them in two dimensions using principal component analysis (PCA; **Figure 1E** and **Figure 1-figure supplement 2D**). The repertoires of naive, central memory, effector, and T regulatory cells are separated by their diversity profiles, and the separation is most pronounced in young mice.

Similar results were observed for the α repertoires, and the diversity of α and β repertoires were highly correlated (**Figure 1-figure supplement 1E**).

In summary, the analysis of the repertoires of different populations captures the known decreasing diversity and increasing clonality of the naive, central memory and effector compartments in both spleen and bone marrow and the decrease in diversity observed with age. These results build further confidence in the reliability of the repertoire sequencing and analysis pipeline.

### Different sub-populations of T cells in young individuals have distinct V gene distributions

**Figure 2:**
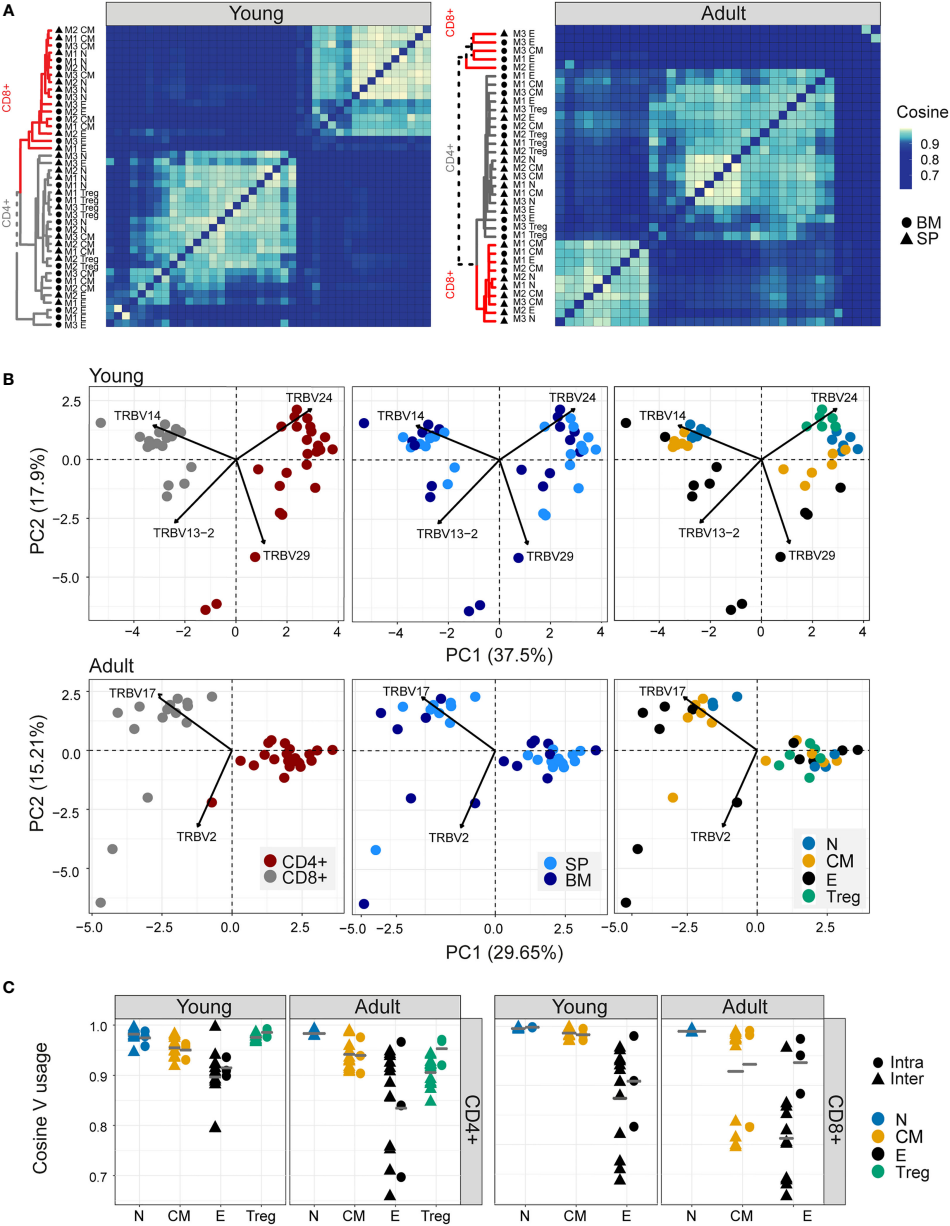
Different sub-populations of T cells in young individuals have different V gene distributions. **(A)** Cosine similarity was calculated between the V gene distributions for all pairs of repertories in young (left) or adult (right) mice and displayed as a heatmap. Hierarchical clustering dendrograms show the assigned organization at each plot, colored by CD4+ and CD8+ groups (grey and red branches respectively) and labels by compartment (text and symbol). Tissues are marked in symbols shape (SP = triangles, BM = circles). **(B)** PCA of the Vβ usage of CD4+ and CD8+ compartments in young (upper) and adult (lower) panels. Each color represents one compartment from one mouse (e.g., CD8+ Effectors, BM, mouse 1). See legend for symbols and color code. The Vβ genes with the highest influence (loading) are marked with arrows. **(C)** Cosine similarity between Vβ gene usage distributions between individuals (circles) or within individuals (between spleen and bone marrow, triangles). The inter-individual variability was calculated separately for spleen and bone-marrow. Each point is the cosine value calculated between two different mice and tissues (SP-SP, SP-BM, BM-BM). T cells compartments (colored dots) are divided into CD4+ (left) and CD8+ (right) from young or adult mice. Mean is shown by horizontal grey lines.

As reported previously (41, 42), both Vα and Vβ gene usage (calculated using the summed UMI abundance for each TCR) was non-uniform in all the repertoires examined, reflecting differential usage of V genes in the recombination process (1) (**Figure 2- figure supplement 1A**). Almost no differences were observed between young and adult naive repertoires. Still, several V genes significantly differed between the experimental repertoires and the synthetic repertoires, reflecting selective pressure during thymic development. The pairwise similarity between V gene distributions of different repertoires was quantified using the cosine similarity between the distributions (see Methods) (**Figure 2A**). We also used the Horn similarity index (29, 43) and found these two measures highly correlated (**Figure 2- figure supplement 1B**).

In young individuals, there was a clear segregation between CD4+ and CD8+ repertoires, and between naive, central memory, effector, and Treg populations, but little distinction between spleen and bone marrow within each sub-compartment. In contrast, the Vβ gene usage in repertoires from older animals was more heterogeneous, especially the repertoires of the CD8+ effector compartments, which diverged between individuals.

PCA on the pairwise similarity matrix for Vβ usage is shown in **Figure 2B**. In young mice, there is a clear separation of both CD4+ and CD8+ repertoires, and of repertoires from different functional compartments, but not between spleen and bone marrow. The Treg populations lie closest to the naive. In adult mice, the separation between CD4+ and CD8+ repertoires is retained, but the distinction between functional compartments largely collapses.

In contrast to the TCRβ repertoires, the equivalent analysis for the α repertoires (**Figure 2- figure supplement 1C, D**) showed much less evidence of consistent structure in either heatmap or PCA. Furthermore, there was only a limited correlation between the cosine similarities of α and β repertoires, especially in the older individuals (**Figure 2- figure supplement 1E**). The selective pressures which shape the repertoires of different CD4+ and CD8+ compartments therefore seem to be reflected differently in Vα and Vβ gene usage.

The intra-individual (spleen versus bone marrow) and inter-individual V gene distribution similarities within each functional population are shown in **Figures 2B, C**. The high variability observed when comparing bone marrow to the spleen is partly a reflection of the very small sample size of the bone marrow samples because, in subsampled repertoires, the inter-individual variation was greater than the intra-individual variation in most cases (**Figure 2- figure supplement 2A**). The plots illustrate a hierarchy of variance, with naive repertoires being closest to each other, followed by central memory and Tregs, and with effector repertoires showing the greatest divergence. The quantification of this hierarchy and its biological meaning is explored in more detail below (**Figure 5**). In contrast, the SONIA-generated synthetic repertoires were very similar to each other (**Figure 2- figure supplement 2B, C**, left panel).

We examined the impact of clonal distribution on inter-repertoire variance. We created a second set of artificial repertoires (control 2), in which we replaced each TCR from a central memory or effector repertoire by a TCR selected randomly from the set of unique TCRs in the combined repertoires of all the mice, but kept its abundance the same. In this way we created new repertoires, with random allocation of TCR sequences, but fixed clonal distributions. The PCA plot of the different populations using control 2 repertoires is shown in **Figure 2- figure supplement 2B, C** (right panel). The separation between subpopulations is largely lost in the controls. However, the inter-repertoire variation of these control CD8+ effectors sets were comparable to those of the real data. The increased inter-repertoire variation in effector repertoires is at least in part attributable to clonal expansion.

### Nucleotide sequence sharing patterns differ between T cell sub-compartments

**Figure 3:**
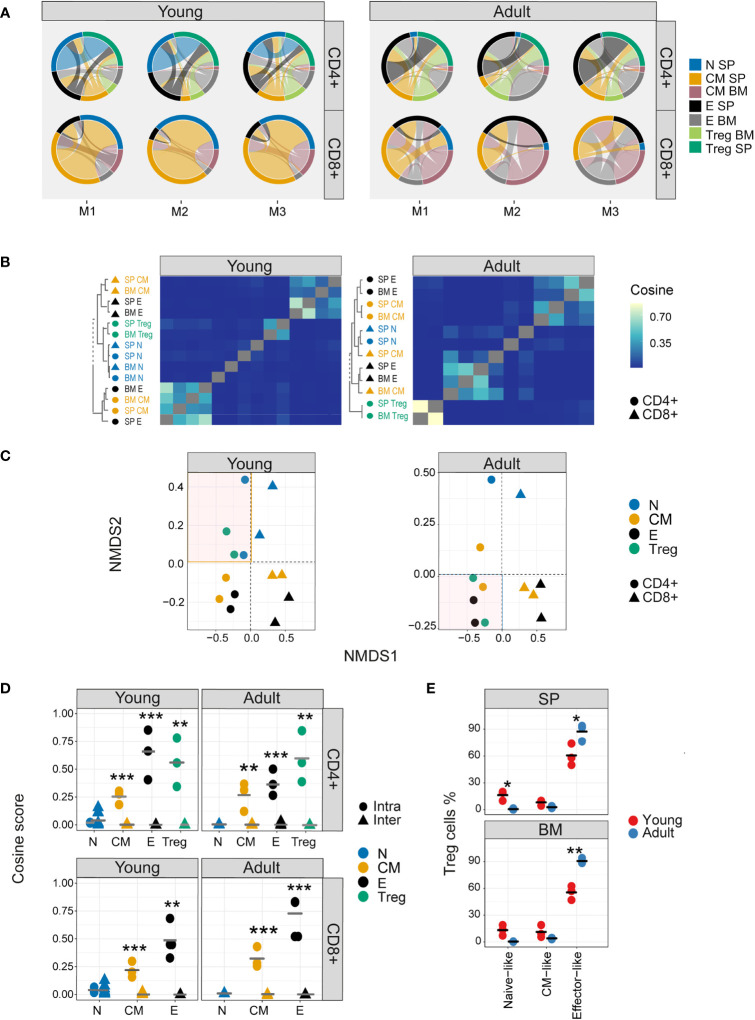
Nucleotide sequence sharing patterns differ between T cell sub-compartments. **(A)** Each circus plot represents a single mouse CD4+ or CD8+ compartment (upper and lower panel, respectively). Circus sharing levels illustrate the number of clones shared between two compartments (band widths), and the proportion of shared clones attributed to each compartment (circus arcs). Only sequences shared by at least two compartments were included in the analysis. **(B)** Pairwise cosine similarity of the CDR3βNT sequences from representative young and adult mouse repertoires. Correlation levels are represented by color (high=light blue, low= dark blue). In color and text, hierarchical clustering dendrograms for all T cell compartments are plotted to the left of each heatmap (CD4+=circle, CD8+= triangles). **(C)** The similarity matrices shown as heatmaps in B are represented in two dimensions by NMDS. **(D)** Cosine similarity between CDR3NTβ chains across (triangles) and within individuals (between spleen and bone marrow, circles). T cells compartments (colored dots) are divided to CD4+ (upper) and CD8+ (lower) from young (left) and adult (right) mice repertories. Mean is shown by horizontal black lines. **(E)** The surface phenotype of Foxp3+ Tregs. The plot shows the percentage of Foxp3 positive cells (Treg) which have the phenotype: CD44- CD62L+ (naive-like), CD44+CD62L+ (central memory -like) or and CD44+CD62L- (effector-like). Mean is shown by horizontal black lines. Each data point represents one mouse. Significant differences between age groups or intra and inter individuals are denoted by asterisks (P-values: *<0.05, **< 0.01, ***< 0.001, with FDR correction t-test).

The TCR V gene distributions analysed above create a simplified abstraction of individual repertoires, and TCR repertoires can also be considered as a hyperdimensional feature space defined by the number of individual nucleotide sequences which constitute each repertoire. We visualized the qualitative patterns of sharing between CD4+ and CD8+ sub-compartments, using circus plots (**Figure 3A**). This analysis, which included only sequences shared by at least two compartments, reveals a distinctive pattern of sharing which is conserved between individuals, and is age specific. In young individuals, CD4+ and CD8+ splenic naive and CD8+ central memory repertoires contribute the highest proportion of shared sequences (blue [0.21-0.26, 0.28-0.39], CD4+ and CD8+, respectively, and orange [0.29-0.39]) circus arc lengths. Naive repertoires from adult mice contribute a much smaller proportion (0.004-0.03, 0.03-0.12, CD4+ and CD8+, respectively) of sharing with other repertoires, and CD4+ (0.307-0.313, 0.12-0.23) and CD8+ (0.195-0.375,0.11-0.23) effectors sequences now make up the largest proportion of shared sequences (blue, black, and grey, circus arc lengths, in SP and BM, respectively). Interestingly, high levels of overlap (0.172-0.307) are observed between young mice splenic CD4+ Treg and CD4+ naive repertoires, while in adult mice, Tregs become more similar to CD4+ effector cells (0.159-0.290). This observation is investigated in more detail below.

Nucleotide sequence sharing was quantified by the pairwise cosine similarity between repertoires. Because the similarity between repertoires of different individuals at nucleotide level is very low, we first analysed each mouse separately. However, visual inspection suggested the patterns obtained for all three mice were very similar, especially for the younger individuals, and this was confirmed by quantitative comparisons of the similarity indices between the different mice (**Figure 3- figure supplement 1A**). A representative heatmap of all pairwise comparisons for a single mouse is shown in **Figure 3B** (TCRβ) and **Figure 3- figure supplement 1B** (TCRα), and the similarity matrix is visualized in two dimension using multidimensional scaling in **Figure 3C** (TCRβ) and **Figure 3- figure supplement 1C** (TCRα). In young mice a hierarchical structure was observed, with naive and Treg repertoires clustered together, and effector and central memory repertoires for CD4+ and CD8+ T cells forming distinct clusters. In older individuals, this structure is perturbed. CD4+ and CD8+ repertoires remain distinct, but Tregs now cluster independently of naive, and are closer to CD4+ effector repertoires. There was modest correlation between TCRα and β similarities, especially in the older individuals (**Figure 3- figure supplement 1D**). The synthetic (control 1) and artificial (control 2) repertoires showed very little sharing or structure (**Figure 3- figure supplement 2**).

The intra-individual (spleen versus bone marrow) and inter-individual nucleotide similarity within each functional population are shown in **Figure 3D** and **Figure 3- figure supplement 1E**. Inter-individual similarity index at nucleotide level is very low in all compartments. The overall intra-individual hierarchy observed is reversed compared to V region usage (**Figure 2C**), reflecting increased overlap of expanded TCR clones shared between spleen and bone marrow in the more differentiated populations. Treg repertoires were more similar to themselves than to other repertoires, but more similar to CD4+ effector repertoires in older than in younger mice (**Figure 3- figure supplement 1E**). The shift from a naive-like to an effector-like Treg observed from the perspective of repertoire sharing was also observed in protein phenotype, with a higher proportion of Foxp3+ CD62L+ CD44- naive-like Tregs in young animals, and a higher proportion of Foxp3+ CD62L-CD44+ effector-like Tregs in the older animals (**Figure 3E**).  

### Differential frequency of amino acid motifs in TCR repertoires from different subpopulations

**Figure 4:**
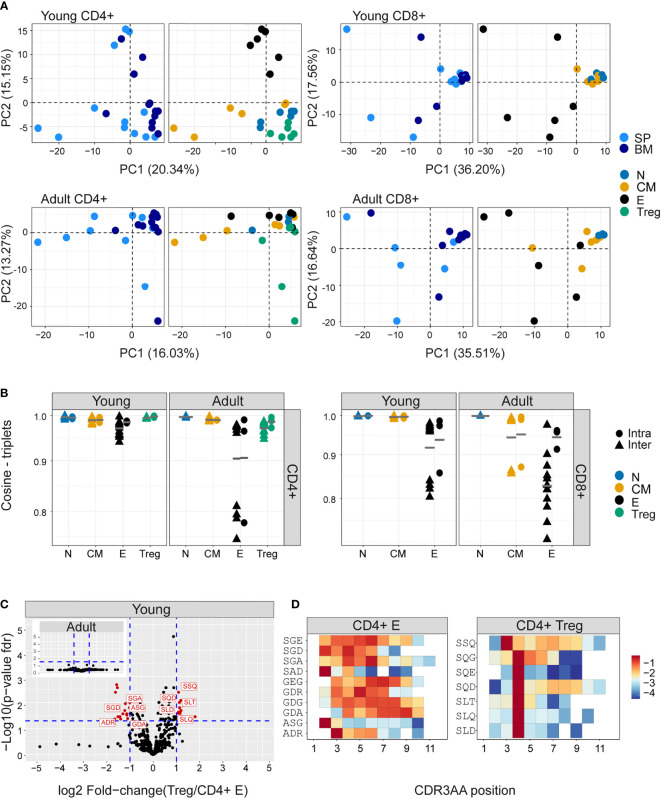
Differential frequency of amino acid motifs in TCR repertoires from different subpopulations. The most abundant amino acid triplets are selected by the mean frequency of each motif across all compartments and mice as described in materials and methods **(A)** PCA analysis of CDR3βAA triplet frequency distributions for CD4+(left) or CD8+(right) from young (upper) or adult (lower) mice (e.g., CD8+ effectors, BM, mouse 1). **(B)** Cosine similarity between the frequency distributions of the 350 most abundant CDR3βAA amino acid triplets between individuals (circles) or within individuals (between spleen and bone marrow, triangles). T cells compartments (colored dots) are divided into CD4+ (left) or CD8+ (right) from young or adult mice. Mean is shown by horizontal black lines. **(C)** Differentially expressed triplets in Treg and CD4+ effector from young and adult mice. Each dot represents a single triplet most 350 abundant or all 8000 triplets in red or black dots, respectively). P-value (t-test) was calculated for each triplet across six samples (three mice and 2 tissues) of CD4+ Treg and CD4+ effector cells. The y-axis shows FDR-adjusted p-values. The x-axis shows the log 2-fold-change, calculated between Treg and CD4+ effector mean triplets or motifs frequency across compartments (6 samples in each). Significance thresholds are marked in blue lines: (1) at y=1.3 (equivalent to p-value of 0.05) and x= ± 1 (denoting a total fold-change of 2). Representative triplets above both thresholds are labeled with red text and dots. **(D)** Significantly expressed triplets are found in various positions along the CDR3AA sequences. Triplets overexpressed in CD4+ Treg are frequently located in position 4 of the CDR3AAs (3–9). Triplets overexpressed in CD4 effector can be located mainly in position 2-3 or further along the CDR3AA sequences. The color represents the log10 frequency of each aligned triplet.

**Figure 5:**
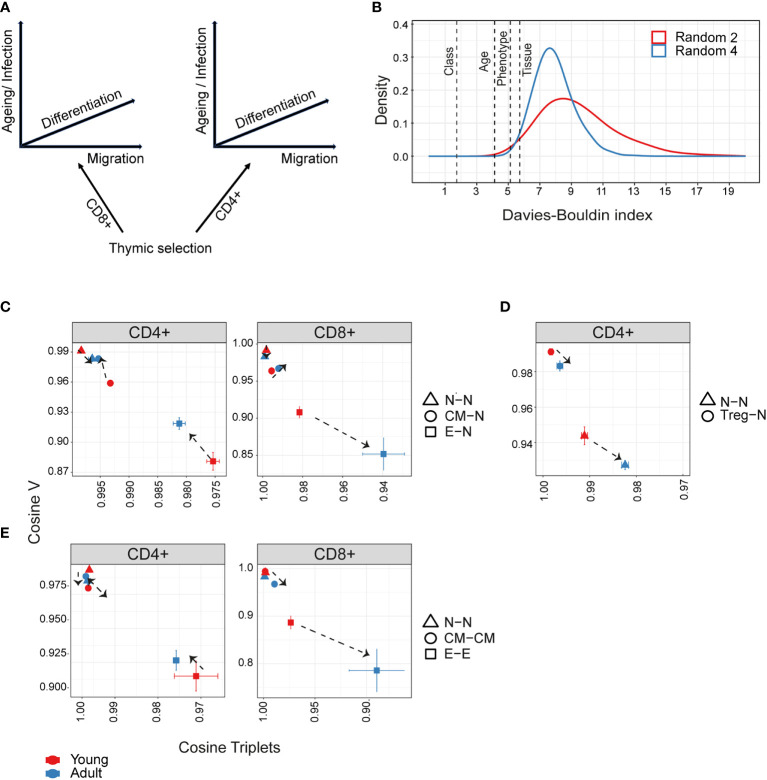
Hierarchical impact of different immunological processes on repertoire diversification. The TCR repertoire is considered as evolving in four dimensions, captured by the diagram in **(A)**. **(B)**The Davies-Bouldin **(DB)** index applied to Vβ frequencies, capturing the average separability (ratio of the within-cluster variance to the separation between cluster centroids (lower score means better clustering)) of clusters of different repertoires from their nearest counterpart. The reference distribution computed by assigning random clusters features (2 or 4 variables, red and blue lines respectively) to the same data and calculating the DB index 10000 times. **(C–E)** The mean inter-repertoire cosine similarity values of the Vβ gene distribution versus the mean inter-repertoire cosine similarity values for the 350 most abundant CDR3βAA triplets. **(C, D)** Each repertoire (spleen only) is compared to the young naive repertoires for CD4+, CD8+ **(C)** and CD4+ Treg **(D)**. In E, each repertoire is compared to each other repertoire from the same compartment. The error bars represent SEM. Arrows show shift from young (red dots) to adult (blue dots).

The extreme hyper-dimensionality of the sequence space dominates individual patterns of clonal diversity and expansion, and limits the recognition of conserved repertoire organization. We and others (38, 44) have shown that short patterns of sequential amino acids (k-mers) can play a key role in determining specificity, and offer one way to reduce the dimensionality of the repertoire while reflecting the complexity of antigen recognition. We therefore counted the presence of sequential amino acid triplets (dimensionality 20^3^) or 7-mers (dimensionality 20^7^) in each repertoire. To further reduce the dimensionality of the feature space, we removed rarely used features (see Methods). The distributions of triplet and 7-mers frequencies are represented in two dimensions by the first two components of a PCA. The k-mer distributions separated CD4+ and CD8+ TCRβ repertoires in both young and older mice (**Figure 4- figure supplement 1A, B**). In the younger repertoires, conserved distinct patterns of k-mer frequency were also evident between the naive, Treg, central memory and effector CD4+ sub-compartments (**Figure 4A** and **Figure 4- figure supplement 1C**), with Tregs lying close to the naive, and central memory repertoires lying between naive and effectors. This hierarchy became more relaxed in the older individuals. Within the CD8+ compartment, central memory and naive cells cluster together, and the overall pattern is driven by a high variance of the CD8+ effectors, which diverge from each other both within and between individuals. A similar qualitative pattern was seen for TCRα triplets and 7-mers, although the distinction between naive and central memory was evident in both CD4+ and CD8+ compartments (**Figure 4- figure supplement 2A, B**). The intra-individual (spleen versus bone marrow) and inter-individual k-mer distribution similarity within each functional population are shown in **Figure 4B** (triplets) and **Figure 4- figure supplement 2C** (7-mers). The plots illustrate a hierarchy of variance, with naive repertoires being closest to each other, followed by central memory and Tregs, and with effector repertoires showing the greatest divergence. The quantification of this hierarchy and its biological meaning is explored in more detail below (**Figure 5**).

We examined in more detail the differential usage of amino acid motifs between Treg and T effectors (**Figure 4C**, **Figure 4- figure supplement 3A**). In younger repertoires ten triplet motifs were over-represented in the CD4+ effector repertoires, and seven in the Treg repertoires. In the older repertoires there was little evidence of differential motif use between these compartments (see insets). Almost all the differentially-represented triplets began with a serine (**Figure 4D**). The triplet motifs over-represented in the Treg repertoires were found almost exclusively at positions 3/4 of the CDR3 suggesting they may be acting as a surrogate for selective V genes; however the triplets over-represented in the T effectors were more broadly distributed across the CDR3 (**Figure 4D**). The 7-mers over-represented in the CD4+ T effectors were predominantly found associated with a single V gene. In contrast, the 7-mers over-represented in Treg repertoires were more broadly distributed (**Figure 4- figure supplement 3B**). Overall, while V gene usage plays a part in the amino acid motif distribution profiles, selection independent of V gene is clearly at work.

### Capturing the relative contribution of different immunological processes to repertoire diversification

We can consider the repertoires we describe above as evolving and diversifying in a multi-dimensional selective space, whose dimensions (selective pressures) include thymic CD4+/CD8+ lineage development, peripheral differentiation (along the naive-memory-effector axis), migration (spleen – bone marrow) and ageing (**Figure 5A**). We quantified the relative contributions of these different processes by combining the global repertoire parameters of V gene and triplet frequency distributions (**Figures 2**, **4**). Our first approach was to measure the separability of repertoires clustered according to each dimension, using the Davies-Bouldin index (DB) (45). The DB index clearly identifies the CD4+/CD8+ division as the most significant driver of repertoire differences as measured by V gene usage (**Figure 5B**), or triplet usage (**Figure 5- figure supplement 1A**). This is consistent with the separation between CD4+ and CD8+ repertoires seen in all the PCAs (**Figures 2**-**4**). The differences between spleen and bone marrow were the least pronounced.

We therefore focused on the influence of ageing and differentiation, analysing CD4+ and CD8+ lineages independently (**Figures 5C-E**, **Figure 5- figure supplement 1B, C**). Diversification of the repertoires is observed as a consistent shift from top left (most similar, naive) to bottom right (most different, effector). The transition from naive to central memory imposes only small changes, and the major change occurs in the transition from central memory to the effector. The interaction between age and differentiation is complex and lineage dependent. Age has only a very minor effect on the organization of the naive repertoires. For CD4+ T cells (**Figure 5C** left panel), the biggest shift between naive and central memory/effector occurs in young mice, and the distributions revert towards naive in adults (note the reverse direction of the arrows in the CD4+ panel). Conversely, in CD8+ cells (**Figure 5C** right panel), age drives additional diversification in both central memory and effector. Treg CD4+ (**Figure 5D**) are quite distinct from naive in both young and adult, but move further from naive with age, as observed in **Figures 2**, **3**.

In **Figure 5E**, we consider diversification not in relation to the naive repertoires but between individual repertoires of the same age and differentiation stage. The most striking observation is that there is a very high level of similarity between inter-individual repertoires of the same stage in either naive or central memory repertoires of both CD4+ and CD8+. In contrast, effector repertoires show a much greater idiosynchratic divergence, which decreases with age for CD4+ T cells, but increases with age for CD8+ repertoires.

We compared these results with equivalent plots (same scale) using the control 2 artificial repertoires (**Figure 5- figure supplement 2A, B**). Most of the structure discussed above is not visible in the controls. The effector control repertoires did show some increase in diversity (**Figure 5- figure supplement 2A**), especially in the CD8+ compartment. Clonal expansion, acting on a random selection of TCRs, therefore drives some stochastic and idiosynchratic diversification of the repertoire.

### The impact of LCMV infection on repertoire organization

**Figure 6:**
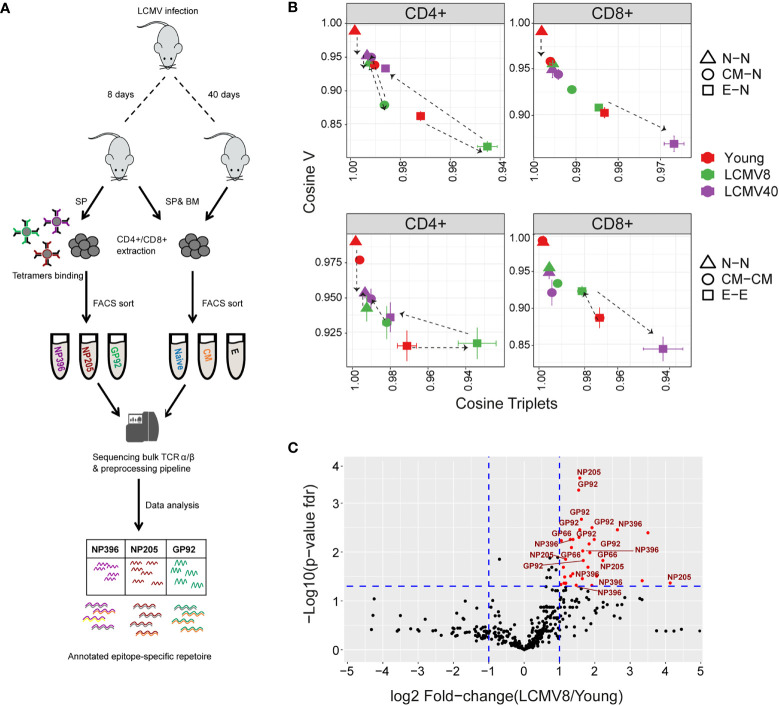
The impact of LCMV infection on repertoire organization. **(A)** Summary of the LCMV induced T cell compartments and epitope –specific cells isolation for TCR repertoire sequencing and analysis. **(B)** The mean inter-repertoire cosine similarity values of the Vβ gene distribution versus the mean inter-repertoire cosine similarity values for the 350 most abundant CDR3βAA triplets. In the upper panel each repertoire (spleen only) from LCMV infected mice is compared to the young naive repertoires for CD4+ and CD8+ compartments. In the lower panel each repertoire (spleen only) is compared to each other repertoire from the same compartment. The error bars represent SEM. **(C)** Triplets over-represented in CD8+ effector repertoires after 8 days of LCMV infection compared to young uninfected healthy mice. Each dot represents a single triplet. The y-axis shows FDR-adjusted t-test p-values. The x-axis shows the log 2-fold-change, calculated between mean triplets from young and LCMV infected mice (6-8 samples in each). Significance thresholds are marked in blue lines: at y=1.3 (equivalent to p-value of 0.05) and x= ± 1 (denoting a total fold-change of 2). Representative triplets above both thresholds are labeled with red text and dots. Significantly enriched triplets that are labeled in red text are also found in the TCRs of epitope specific (NP396, NP205, and GP92 tetramer sorted) T cells.

The differentiation of memory and effector populations in the healthy mice kept in a specific pathogen-free (SPF) environment is believed to be due to low level exposure to a set of self-and commensal derived antigens present in the animal house environment. As a contrast, we examined the effect of exposure to a strong acute immunogenic stimulus on the organization of the immune repertoires (**Figure 6A**). We infected C57BL/6 mice with LCMV, which drives a strong but self-limiting infection associated with a well-characterized immune response.

The cosine similarity for each compartment between mice, as well as between repertoires of young and older uninfected individuals, is shown for the V gene, CDR3 nucleotide, and amino acid triplets (**Figure 6- figure supplement 1A**). Infection drives strong changes in the V gene and triplet distributions. We plotted the impact of LCMV infection on the repertoire using the same framework shown in **Figures 5C, E** (**Figure 6B**). For greater clarity, the mean for each set of comparisons is shown in **Figure 6B**, while individual pairwise comparisons for TCRβ are shown in **Figure 6- figure supplement 1B**. Equivalent plots for TCRα are shown in **Figure 6- figure supplement 1C**. Acute LCMV infection (LCMV8) drives naive, central memory, and effector CD4+ repertoires to diverge both from the uninfected naive repertoire and from each other. In most cases, the repertoires return towards their pre-infection state by day 40. In the CD8+ compartment, acute LCMV infection also drives increases in naive and central memory diversity, albeit less than those observed in CD4+. However, acute infection drives an increased similarity in the effector compartment (**Figure 6B**), consistent with a narrowed repertoire produced by large expansions of a set of sequence-related TCRs, as observed in **Figure 6C**.

The antigen-driven effect in aged and LCMV infected mice was validated by increased levels of coding-degeneracy levels in splenic CD8+ effector cells (46) (**Figure 6- figure supplement 2**).

In order to understand better the convergence observed between the effector populations of infected mice, we analysed triplet usage in the CD8+ effectors of LCMV infected versus uninfected individuals. 36 triplet motifs were highly enriched in the repertoires of the LCMV infected mice (**Figure 6C**, sequences in **SI Table 2**). Remarkably, all these triplets were also observed in the TCRs of a population of T cells isolated from the infected spleens by sorting on the LCMV peptides NP396-404(H-2Db), NP205-212(H-2Kb) and GP92-101(H-2Db) (**Figure 6A**). In contrast, a random same size set of non-enriched triplet motifs showed significantly less (26/36, Fisher’s test p=0.0009) overlap with the triplets observed in the epitope sorted TCRs. LCMV infection therefore drives expansion of a set of TCRs which share a limited number of triplet motifs, thus driving a temporary convergence of the TCR repertoire in the CD8+ effector populations.

## Discussion

The adaptive immune system, uniquely among vertebrate physiological systems, uses a family of receptors which are not encoded in the germline, but are created *de novo* in each individual by a stochastic process of imprecise DNA recombination. A fundamental task for immunologists is to understand how this stochasticity and associated inter-individual heterogeneity can nevertheless result in a robust and regulated response to an enormous diversity of antigens in most individuals of a population. In this study, we explore the balance between stochasticity and heterogeneity on the one hand and order and consistency on the other. We systematically analyze the TCR repertoire of different functional and anatomical compartments of the adaptive immune system, sampled from young (3 months) and adult (12 months) mice. These repertoires capture the influence of multiple selective pressures, including thymic lineage selection (CD4+/CD8+), peripheral differentiation (along the naive-memory-effector axis), migration (spleen – bone marrow), aging, and infection (illustrated in **Figure 5A**). We document the effects of these selective processes on different features of the repertoire, which span the range from the full hyper-dimensionality of individual nucleic acid sequences (>10^8^ per mouse) through the enumeration of amino acid motifs (a few hundred), to the frequency of different V genes (20). We focus the analysis on quantitative measurements of similarity between repertoires, which reflects both convergent and divergent evolution of the repertoire. A recent study has reported systematic sequencing of the TCR repertoire of different human T cell subsets, but the focus of their analysis was on the biochemical characteristics of the TCR (47). In all our analyses, we measure similarity between repertoires using metrics that incorporate clonal expansion and do not compare only unique sets of TCRs. We believe this is an essential characteristic of the analysis. Antigens exposure and differentiation drive both selection and expansion – in fact, selection and expansion are really one process which together drive repertoire evolution. Comparisons which ignore clonal expansion do not capture the underlying biology of the processes we seek to study.

Thymic selection of distinct CD4+ and CD8+ lineages has the strongest impact on the structure of the repertoire, as shown both by the clear demarcation seen between CD4+ and CD8+ compartments in the PCAs, and by the analysis of the Davies-Bouldin clustering index. The effect of this selection on each individual feature is subtle, and the overall effect cannot be captured by any single feature (V gene, triplet, etc.). However, the impact is remarkably resilient to the effects of antigen exposure, both from the perspective of age and functional differentiation, and clonal expansion. In contrast, the anatomical origin of the repertoires has the most subtle effects on structure, except for the exclusion of naive cells from the repertoire.

Age and differentiation status had an intermediate effect on the repertoire structure. PCA analyses of the V gene and k-mer frequency distributions showed a gradual relaxation of the overall structure with age, with less clear demarcation between different functional compartments in the adult mice than in the young mice. However, a careful quantitative analysis of these global parameters revealed that the impact of differentiation and age on the repertoire structure was subtle and complex and was influenced by CD4+/CD8+ lineage. In both CD4+ and CD8+ T cells, there is a gradual diversification of the repertoire, away from the naive and also away from each other, as one moves along the naive to central memory to the effector axis, with the shift to effector having the strongest overall effect. In part, these effects can be explained in terms of increasing clonal expansion, which allows some individual TCR sequences to affect the global repertoire properties. However, this explains only some of the effects, and more complex models involving a selection of particular TCR features (V gene, k-mer, etc.) will be required to explain the data. Age has a minimal effect on the global parameters of N repertoires, reflecting the stability of the generation of diversity mechanisms in young and adult individuals. In the CD4+ compartment, central memory and effector divergence from naive is most pronounced in the young mice, presumably reflecting early exposure to environmental antigens, and the parameters return towards naive and each other in the adult. In the CD8+ compartment, in contrast, central memory and effector diverge progressively as the mice age, perhaps reflecting chronic antigen exposure. These selective forces must operate on the TCRα/β heterodimer since the two genes are co-expressed as a single structure at the cell surface. However, the selection seems to operate rather independently on the α, and β sequences since the patterns of inter-repertoire sharing observed for α and β are only loosely correlated. Vβ genes are much more informative than Vα genes in terms of distinguishing functional compartments. Interestingly, analysis of V region usage and k-mer usage gave rather similar overall patterns and hierarchy of repertoire structure and evolution. This is not intuitive, since V gene usage is driven by recombination biases and MHC interactions, while CDR3 triplet usage is believed to reflect antigen specificity. The fact that we see a similar hierarchical pattern when exploring two different parameters is an important observation that suggests mechanistic integration of these different processes to preserve the structure of the repertoire. The molecular basis of these effects remains unclear but provides an exciting challenge for future experimental investigation.

The tension between randomness and directed evolution is most evident when comparing the analysis of V gene frequencies and individual CDR3 nucleotide sequences. The similarity in V gene usage is greatest in naive and decreases progressively in central memory and effector repertoires. In contrast, similarity in CDR3 frequencies is lowest in naive because of the extreme diversity of this compartment and increases progressively in central memory and effector repertoires. The combination of recombination and selection, therefore, imposes a rigid pattern of V gene usage, which nevertheless encompasses an enormous diversity of TCR sequences. Memory and effector differentiation, presumably in response to antigen, drive some convergent evolution of the clonal repertoire, reflected by increasing similarity of nucleotide sequence repertoires. In combination with increasing clonal expansion, which allows small number of individual clones a disproportionate influence on the overall repertoire, this increasingly disturbs the rigid pattern of V gene usage.

The Treg population shows a distinctive distribution of similarities. In both young and adult mice, the Treg repertoires are more similar to themselves than to any other compartment, confirming the distinct nature of the Treg repertoire, which has been hypothesized to arise from exposure to a distinct set of antigens (48, 49). However, the Treg repertoires are more similar to naive repertoires in the younger individuals but become more similar to effector repertoires with age. The switch from a naive-like to a more effector-like repertoire, which is also observed at a phenotypic level by increased expression of CD44 and decreased expression of CD62L may reflect life-long gradual recruitment of induced Tregs to the original natural Treg population emerging from the thymus (50). The switch of regulatory T cells to a more effector phenotype might also represent a weakening of regulatory activity and hence be linked to the increase in autoimmunity associated with age.

The response to environmental antigens drives many of the differentiation and age-associated changes which we describe. Since the mice are housed in specific pathogen-free conditions and are not germ-free, this may include a variety of microbial antigens present in the environment. However, although the mice are co-housed, the individual antigen exposure may be heterogeneous and asynchronous. We, therefore, investigated the impact of exposure to a strong synchronous exogenous antigenic stimulus by infecting the mice with LCMV, which produces a strong but self-limiting infection in the C57BL/6 strain. The immune response to this virus has been studied extensively (51) and is known to involve strong systemic clonal expansion by both CD4+ and CD8+ T cells. Indeed, as expected, the repertoires at 8 days post-infection, when the immune response is strongest (52, 53), showed evidence of perturbation. Interestingly, LCMV induced a marked decrease in similarity in both V gene and amino acid motif usage in both CD4+ and CD8+ naive repertoires, perhaps reflecting increased turnover and perturbation of this compartment in response to the infection. However, in contrast to the changes observed in response to chronic environmental antigen stimulation, LCMV drove an increased similarity of effector repertoires. This was reflected not only in the V gene and CDR3 nucleotide distributions but was evidenced by the existence of amino acid triplets highly enriched in the TCR repertoire of infected individuals. Remarkably, many of these triplets were found within the set of CDR3s of CD8+ TCRs, which bound one specific epitope of LCMV, confirming the link between motifs and specific antigen recognition. Thus, exposure to a strong synchronous source of antigen, such as is provided by acute exposure to LCMV, drives strong convergent evolution and decreased diversity of the TCR effector repertoire, which relaxes partially towards the uninfected state at 40 days post-infection.

The study we present has a number of limitations. The number of individuals analysed was small, limiting the amount of robust statistical analysis which can be carried out. Thus, many of the conclusions we make are based on statistical trends rather than classical statistical significance thresholds. An interesting and potentially fruitful approach to increase statistical power would be to develop more sophisticated mechanistic models of the differentiation and aging processes (as has been explored in (54, 55)), which would capture the key parameters identified in this paper, and allow simulation of multiple synthetic repertoires with similar properties to the data. A further limitation was that the analysis of the effects of aging is limited to two-time points and would benefit from an extension to very young or very old mice. We also recognize that the functional sub-compartments we define are based on a rather simplistic and limited panel of antibody markers, and that in reality the populations we refer to as naive, central memory and effector certainly contain further heterogeneity which could be explored further in future studies.

In conclusion, we present a novel approach to the analysis of the TCR repertoire, which we use to address the fundamental relationship between stochastic and deterministic processes which drive the evolution of the adaptive repertoire. The adaptive immune system shows a remarkable capability to preserve a high-order structure, as reflected by conserved frequency distributions of the V gene and short amino acid linear motifs, while still allowing enormous diversity at individual sequence level. This high-order structure is partially preserved but gradually weakened as the adaptive immune system ages. We speculate that this structure is key to maintaining a robust, consistent antigen-specific response across a population in the face of the randomness and heterogeneity imposed by the process of imprecise TCR recombination.

## Data availability statement

The datasets presented in this study can be found in online repositories. The names of the repository/repositories and accession number(s) can be found below: https://www.ncbi.nlm.nih.gov/, PRJNA771880 https://datadryad.org/stash, https://doi.org/10.5061/dryad.51c59zw96.

## Ethics Statement

The animal study was reviewed and approved by Council for experiments on animals (IACUC) Weizmann institute.

## Author contributions

MM designed the study, prepared and analyzed the data, and wrote the manuscript. SR: 1,2,4. EG: 3,4. DR: 3. AM: 4. BC: 1,3,4. NF: 1,3,4. Contributed with: 1. Design and conception of the study 2. Experimental preparation of the data 3. Data analysis 4. Writing the manuscript. All authors contributed to the article and approved the submitted version.

## Funding

BC was supported by a Weston Visiting Professorship from the Weizmann Institute of Science, and by a grant from the Rosetrees Foundation, UK. NF was supported by the Applebaum Family Foundation.

## Acknowledgments

This study was initiated and conceived by our friend, mentor, and colleague Dr. Nir Friedman (last author). Sadly, Nir died after a long battle with illness without being able to complete the work. We have tried to complete this study in the spirit in which it was undertaken, but we are conscious that we fall far short of the insight and clarity of Nir’s remarkable intellect. We dedicate this study to his memory.

## Conflict of Interest

The authors declare that the research was conducted in the absence of any commercial or financial relationships that could be construed as a potential conflict of interest.

## Publisher’s note

All claims expressed in this article are solely those of the authors and do not necessarily represent those of their affiliated organizations, or those of the publisher, the editors and the reviewers. Any product that may be evaluated in this article, or claim that may be made by its manufacturer, is not guaranteed or endorsed by the publisher.

## Supplementary material

Supplementary Figure 1.1
**(A)** Representative sorting gates for CD4+ cells of one young mouse. **(B)** FACS-sorting cells percentage of each compartment of young (red) or adult (blue). The mean is shown in black lines (n=3). Significant differences between age groups are denoted by asterisks (P-values: *<0.05, **< 0.01, ***<0.001, t-test). **(**C) The number of obtained UMIs and CDR3NTs, correlates with sorted cells number. Dots correspond to the sum of UMI count or CDR3NT number versus the sorted cell number (upper and lower panels, respectively) or the CDR3NT number versus the sum of UMI count (middle panel). The color reflects the tissues or T cell states from TCRα and TCRβ (left and right panels, respectively) sequences from young mice. P-values= 4.46x10^-18^- 1.83x10^-42^, R2 = 0.86-0.97. **(D)** High correlation between TCRα and TCRβ UMI counts. Colored dots correspond to the sum of the UMIs for each repertoire from young mice (color and shape). **(E)** Shannon indices from TCRα and TCRβ repertoires are highly correlated. Each point is the Shannon index of one SP or BM, CD4+, or CD8+ (dots shape) repertoire from young or adult mice.

Supplementary Figure 1.2 Clonal expansion and diversity of the TCRβ repertoire in different bone marrow subsets of young and adult mice. **(A)** The TCRs in each repertoire were ranked according to abundance. The proportion within each decile is illustrated (low abundance sequences in white, ranging to high abundance sequences in dark red). The distribution percentage represented by the top decile is shown in white text. **(B)** The sequence abundance distribution in each compartment. The plots show the proportion of the repertoire (y-axis) made up of TCR sequences observed once, twice, etc. (x-axis). Repertoires from young mice are shown with red dots, older mice with blue dots, and synthetic repertoires in green. **(C)** Simpson and Shannon of subsampled repertoires of equal size (500 CDR3NTs) from each compartment and mouse. Colors same as panel B. Mean is shown in black lines (n=3). **(D)** PCA of the Renyi diversities of order 0, 0.25, 0.5, 1, 2, 4. CD4+ or CD8+ T cells compartments (color dots) from young or adult (left or right panel respectively).

Supplementary Figure 1.3 TCRβ normalized and cumulative frequency distributions in different subsets of young and adult mice. Repertoires from young mice are shown with red dots, older mice with blue dots, and the control 1 repertories in green. **A-B)** The normalized sequence abundance (“UMI count norm” the abundance is divided by the total UMI count for that repertoire) distribution in splenic (A) or bone-marrow (B) compartment. **C-D)** The cumulative abundance distribution in splenic (C) or bone-marrow (D) compartments.

Supplementary Figure 2.1
**(A)** TRV usage of naive cells from young (red), adult (blue), and synthetic (green) mice. Each bar represents the mean frequency of the V segment in the grouped naive T cells from both tissues. Error bars are SEM (n=6, three mice from CD4+ and CD8+ naive). Significant differences between all pair groups (Young vs. Adult= orange, Young vs. Syn=black, Adult vs. Syn=grey) in specific segments are detected both in TRBV genes and TRAV families of genes (P-values: *<0.05, **< 0.01, t-test with Benjamini & Hochberg correction). **(B)** Correlation between Cosine and Horn similarities for TRBV usage. Each point is the pairwise Horn or Cosine score for all compartments. **(C)** The cosine similarity index of the TRAV usage was calculated between all pairs of repertoires in young (left) or adult (right) mice. Hierarchical clustering dendrograms show the organization of the assigned at each plot, colored by CD4+ and CD8+ groups (grey and red branches respectively) and labels by compartment (text and symbol). Tissues are marked in symbols shape (SP= triangles, BM= circles). **(D)** PCA of pairwise cosine similarities for TCR Vα usage. Each color represents one compartment from one mouse (e.g., CD8+ Effectors, BM, mouse 1). **(E)** Correlation between pairwise cosine similarities for TCRVα and TCRVβ gene usage.

Supplementary Figure 2.2
**(A)** The impact of repertoire size on TCR V region similarity. TCR repertoires were subsampled as described in detail in Methods. The subsampling was repeated 100 times, and cosine similarity mean values in each metric were calculated. Pairwise cosine similarities of the TCRVβ usage calculated using all TCRs (blue) were plotted against pairwise cosine similarities of the TCRVβ usage calculated using equal numbers of subsampled TCRs (yellow). The inter-individual variability was calculated separately for spleen and bone-marrow. Each point is the cosine value calculated between two different mice and tissues (SP-SP, SP-BM, BM-BM). T cells compartments (colored dots) are divided into CD4+ (left) and CD8+ (right) from young or adult mice. Mean is shown by horizontal black lines. **(B-C)** Cosine similarities between TCRVβ frequency distributions in control repertoires. (B) PCA analysis of the TCRVβ usage distributions. Each color represents one compartment from one control repertoire (e.g., CD8+ Effectors, BM). See legend for symbols and color code. C) Pairwise cosine similarity scores between TCRVβ usage distribution in different control repertoires. Cosine scores between individuals (circles) or within individuals (between spleen and bone marrow, triangles). Each point is the cosine value calculated between two different repertoires (SP-SP, SP-BM, BM-BM). T cells compartments (colored dots) are divided to CD4+ (left) and CD8+ (right). Mean is shown by horizontal black lines.

Supplementary Figure 3.1 Differential sharing of T cell CDR3 nucleotide α and β chain sequences in different subpopulations of T cells. **(A)** Cosine similarity for CDR3NT between all pairs of compartments within each young or adult mouse (for example, in young mouse 1: Treg SP and CD4+ N BM). These values were compared across mice using another Cosine score calculation. The color corresponds to the TCR chain (red= TCRα, grey= TCRβ). Significant differences between age groups are denoted in asterisks (P-values: *<0.05, **< 0.01, t-test). **(B)** Pairwise cosine similarity from representative young, adult, or control (“Control 1/2”) mouse CDR3αNT sequences. Correlation levels are represented by color (high=light blue, low= dark blue). In color and text, hierarchical clustering dendrograms for all T cell compartments are plotted to the left of each heat map (CD4+=circle, CD8+= triangles). **(C)** The similarity matrices shown as heatmaps in B are represented in two dimensions by NMDS. **(D)** CDR3αNT versus CDR3βNT cosine similarities between all pairwise compartments of young and adult mice. **(E)** Cosine index sharing levels between CDR3βNT of Tregs across tissues or naive and CD4+ effector repertoires within each young(red), adult(blue), or synthetic-based (green) mouse. Comparisons between the different tissues (SP-SP, SP-BM, BM-BM, n= 9). Mean is shown by horizontal black lines. Significant differences are denoted in asterisks (P-values: *<0.05, **< 0.01, t-test) and calculated between the groups: Tregs across tissues and Treg CD4+ naive cells.

Supplementary Figure 3.2 Cosine similarities between CDR3βNT frequency distributions in control repertoires. **(A)** Pairwise cosine similarity scores between CDR3βNT distributions in different control repertoires. Cosine scores between individuals (circles) or within individuals (between spleen and bone marrow, triangles). Each point is the cosine value calculated between two different repertoires (SP-SP, SP-BM, BM-BM). T cells compartments (colored dots) are divided to CD4+ (left) and CD8+ (right). Mean is shown by horizontal black lines. **(B)** Pairwise cosine similarity from control 1 or 2 mouse CDR3βNT sequences. Correlation levels are represented by color (high=light blue, low= dark blue). In color and text, hierarchical clustering dendrograms for all T cell compartments are plotted to the left of each heat map (CD4+=circle, CD8+= triangles). **(C)** The similarity matrices shown as heatmaps in B are represented in two dimensions by NMDS.

Supplementary Figure 4.1 PCA analysis of the frequency distributions of the most abundant CDR3βAA triplets and 7-mers. In panels A, B, the distributions are coloured according to CD4+ or CD8+ lineage. In panel C, CD4+ and CD8+ repertoires are plotted separately, and the individual points are coloured by tissue of origin or subpopulation.

Supplementary Figure 4.2
**(A)**PCA analysis of the frequency distributions of the most abundant CDR3αAA triplets and 7-mers. **(B)**PCA analysis of the top CDR3αAA triplets. PCA analysis of the top 7-mers. CD4+ and CD8+ repertoires are plotted separately, and the individual points are coloured by tissue of origin or subpopulation. **(C)** Pairwise cosine similarities scores of the top 7-mers CDR3βAA motifs between individuals (circles) or within individuals (between spleen and bone marrow, triangles). T cells compartments (colored dots) are divided into CD4+ (left) and CD8+ (right) from young or adult mice. Mean is shown by horizontal grey lines.

Supplementary Figure 4.3 Differential CDR3βAA 7mer frequency in CD4+ Treg and CD4+ Teff TCRs. **(A)** Each dot represents a single CDR3βAA 7-mer motif. P-value (t-test) was calculated for each motif across six samples (three mice and two tissues) of CD4+ Treg and CD4+ effector cells. The Y-axis shows FDR-adjusted p-values. The X-axis shows the log 2-fold-change, calculated between Treg and CD4+ effector mean motifs frequency across compartments (6 samples each). Significance thresholds are marked by the blue lines at y=1.3 (equivalent to a p-value of 0.05) and x= ± 1 (denoting a total fold-change of 2). Representative 7-mers above both thresholds are labeled with red text and dots. **(B)** The TCRVβ usage of the CD4+Treg (right) and CD4+ effector (left) differentially expressed 7-mers. The color represents the log10 frequency of each 7-mer in a specific Vβ gene (low= blue, high=red).

Supplementary Figure 5.1
**(A)** The Davies-Bouldin (DB) index applied to CDR3βAA top triplet motifs, capturing the average separability (ratio of the within-cluster variance to the separation between cluster centroids (lower score means better clustering) of clusters of different repertoires from their nearest counterpart. A reference distribution adding random clustering features (2 or 4 variables, red and blue lines respectively) to the same data and repeated the DB index calculation 10000 times. **(B-C)** V gene similarity plotted against CDRAA top triplet similarity distribution in young vs. adult or young. The β chain sequences of all pair cosine values in B. Mean cosine values for α chain sequences in C. Error bars are SEM. Comparing each repertoire to young naïve in CD4+, CD8+ and CD4+ Treg in young or adult mice (red dots = young mice, blue dots = adult mice).

Supplementary Figure 5.2 TCRVβ gene similarity from control 2 repertoires plotted against CDR3βAA top triplet similarity distribution, comparing each repertoire to young naive in CD4+, CD8+ and CD4+ Treg in young and adult mice (red and blue dots). Each point is the mean cosine score, and the error bars are SEM.

Supplementary Figure 6.1
**(A)** Cosine similarity index of TRBV genes, CDR3βNT, and top CDR3βAA 3-mers (top 350) motifs calculated between tissues and individuals. Colored dots reflect the mice groups (red = young, blue = adult, green/purple = mice after 8 and 40 days of acute LCMV infection, respectively). Horizontal black lines show the mean. The CD4+ and CD8+ naïve repertoires were subsampled as described in detail in Methods. The subsampling was repeated 100 times, and the mean values of each cosine similarity metric were calculated for TRBV genes, CDR3βNT, and top CDR3βAA triplets (350) motif distributions. **(B-C)** TCRV gene distribution similarity plotted against CDRAA top triplet similarity distribution in young vs. LCMV infected mice. The similarity between each repertoire and the young naïve in CD4+ and CD8+ repertoires to day 8 or 40 post LCMV infection (green or purple dots, respectively). The β chain sequences of all pair cosine values in B. Mean cosine values for α chain sequences in C. Error bars are SEM.

Supplementary Figure 6.2 Increased convergent recombination levels in CD8+ splenic effector cells with age and LCMV infection. Each point represents the number of CDR3NT_VJ which encodes for the same CDR3AAβ sequence (convergent recombination or coding-degeneracy) versus the frequency levels in young, adult, and mice after 8 days of LCMV infection (red or blue or green dots respectively).

## Abbreviations

TCR: T cell receptor, BM: Bone marrow, SP: Spleen, CDR3: Complementarity determining region three, CDR3NT/CDR3AA: Nucleotide and amino acid sequences of the CDR3, k-mer: CDR3AA triplets or 7-mers motifs, UMI: Unique molecular identifier, Treg: Regulatory T cells, V, D and J: Variable (V), diversity (D) and joining (J) TCR gene segments, LCMV: Lymphocytic choriomeningitis virus.
