# Supplementary figures and images for: A hierarchy of selection pressures determines the organization of the T cell receptor repertoire
Source: Front Immunol. 2022 Jul 29;13:939394. doi: 10.3389/fimmu.2022.939394 (PMC9372880; doi:10.3389/fimmu.2022.939394)

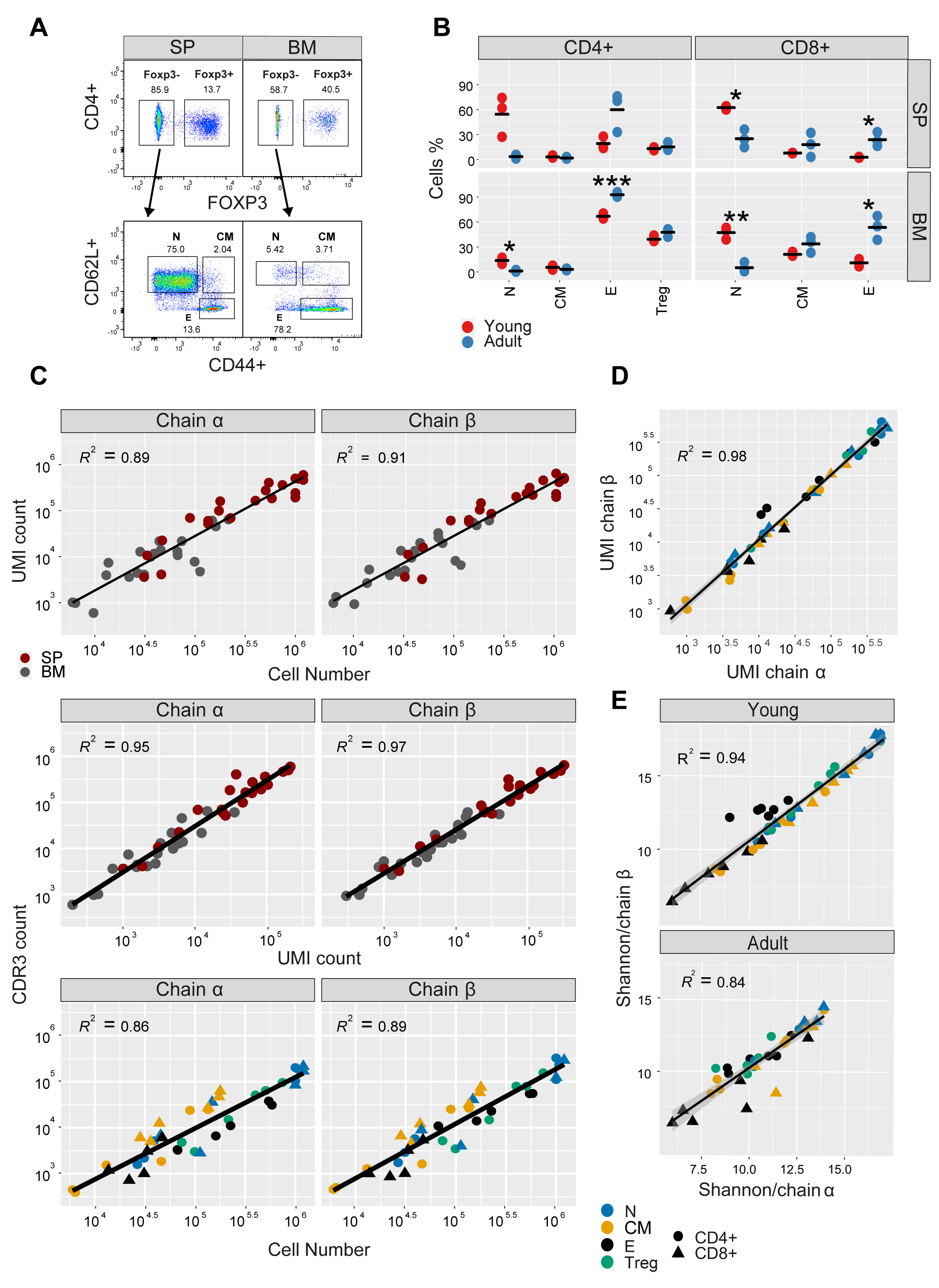

Supplement: Supplementary Figure 1.1 — (A) Representative sorting gates for CD4+ cells of one young mouse. (B) FACS-sorting cells percentage of each compartment of young (red) or adult (blue). The mean is shown in black lines (n=3). Significant differences between age groups are denoted by asterisks (P-values: *<0.05, **< 0.01, ***<0.001, t-test). (C) The number of obtained UMIs and CDR3NTs, correlates with sorted cells number. Dots correspond to the sum of UMI count or CDR3NT number versus the sorted cell number (upper and lower panels, respectively) or the CDR3NT number versus the sum of UMI count (middle panel). The color reflects the tissues or T cell states from TCRα and TCRβ (left and right panels, respectively) sequences from young mice. P-values= 4.46x10^-18^- 1.83x10^-42^, R2 = 0.86-0.97. (D) High correlation between TCRα and TCRβ UMI counts. Colored dots correspond to the sum of the UMIs for each repertoire from young mice (color and shape). (E) Shannon indices from TCRα and TCRβ repertoires are highly correlated. Each point is the Shannon index of one SP or BM, CD4+, or CD8+ (dots shape) repertoire from young or adult mice. [file Image_1.tif]

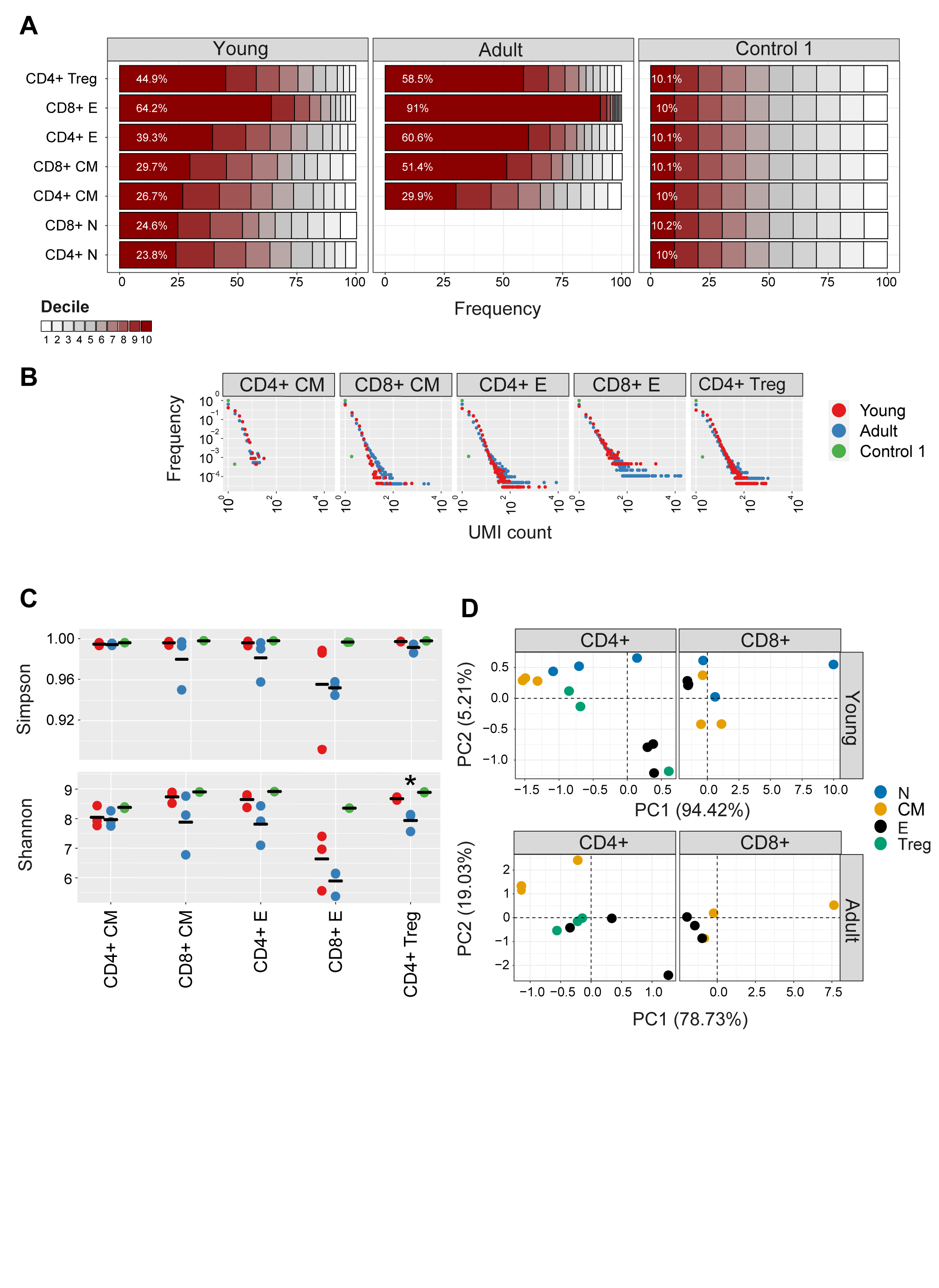

Supplement: Supplementary Figure 1.2 — Clonal expansion and diversity of the TCRβ repertoire in different bone marrow subsets of young and adult mice. (A) The TCRs in each repertoire were ranked according to abundance. The proportion within each decile is illustrated (low abundance sequences in white, ranging to high abundance sequences in dark red). The distribution percentage represented by the top decile is shown in white text. (B) The sequence abundance distribution in each compartment. The plots show the proportion of the repertoire (y-axis) made up of TCR sequences observed once, twice, etc. (x-axis). Repertoires from young mice are shown with red dots, older mice with blue dots, and synthetic repertoires in green. (C) Simpson and Shannon of subsampled repertoires of equal size (500 CDR3NTs) from each compartment and mouse. Colors same as panel B. Mean is shown in black lines (n=3). (D) PCA of the Renyi diversities of order 0, 0.25, 0.5, 1, 2, 4. CD4+ or CD8+ T cells compartments (color dots) from young or adult (left or right panel respectively). [file Image_2.tif]

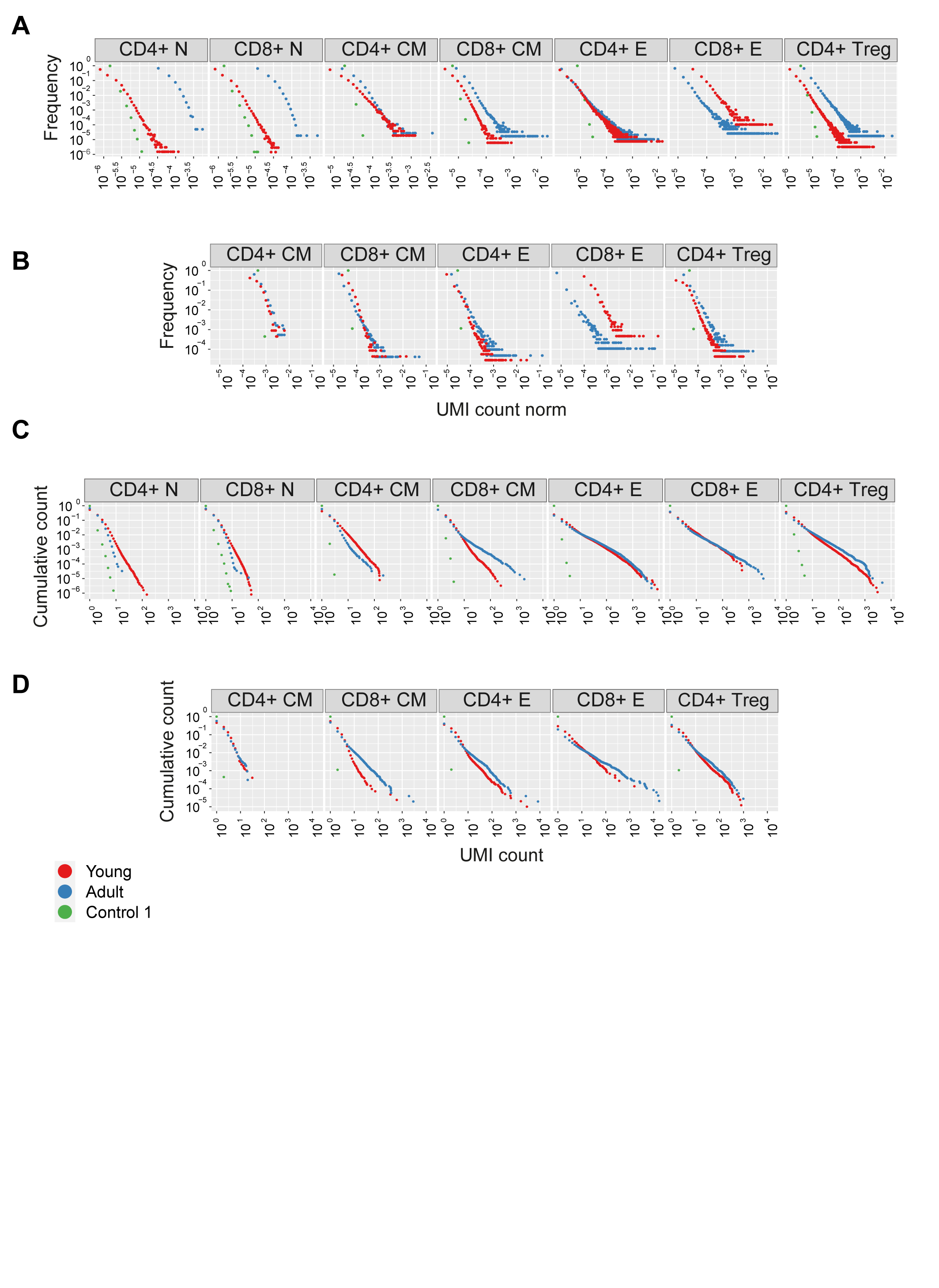

Supplement: Supplementary Figure 1.3 — TCRβ normalized and cumulative frequency distributions in different subsets of young and adult mice. Repertoires from young mice are shown with red dots, older mice with blue dots, and the control 1 repertories in green. A-B) The normalized sequence abundance (“UMI count norm” the abundance is divided by the total UMI count for that repertoire) distribution in splenic (A) or bone-marrow (B) compartment. C-D) The cumulative abundance distribution in splenic (C) or bone-marrow (D) compartments. [file Image_3.tif]

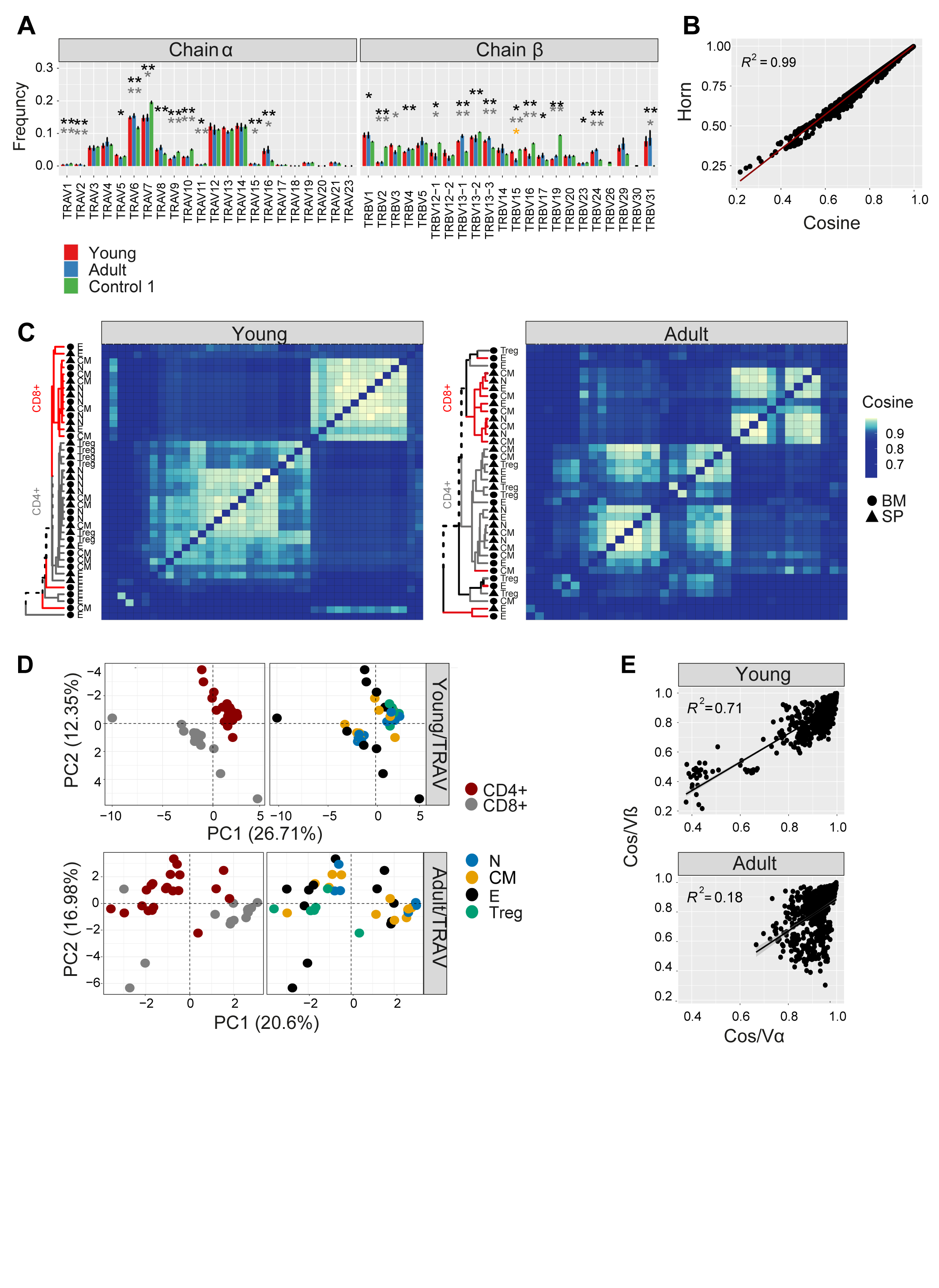

Supplement: Supplementary Figure 2.1 — (A) TRV usage of naive cells from young (red), adult (blue), and synthetic (green) mice. Each bar represents the mean frequency of the V segment in the grouped naive T cells from both tissues. Error bars are SEM (n=6, three mice from CD4+ and CD8+ naive). Significant differences between all pair groups (Young vs. Adult= orange, Young vs. Syn=black, Adult vs. Syn=grey) in specific segments are detected both in TRBV genes and TRAV families of genes (P-values: *<0.05, **< 0.01, t-test with Benjamini & Hochberg correction). (B) Correlation between Cosine and Horn similarities for TRBV usage. Each point is the pairwise Horn or Cosine score for all compartments. (C) The cosine similarity index of the TRAV usage was calculated between all pairs of repertoires in young (left) or adult (right) mice. Hierarchical clustering dendrograms show the organization of the assigned at each plot, colored by CD4+ and CD8+ groups (grey and red branches respectively) and labels by compartment (text and symbol). Tissues are marked in symbols shape (SP= triangles, BM= circles). (D) PCA of pairwise cosine similarities for TCR Vα usage. Each color represents one compartment from one mouse (e.g., CD8+ Effectors, BM, mouse 1). (E) Correlation between pairwise cosine similarities for TCRVα and TCRVβ gene usage. [file Image_4.tif]

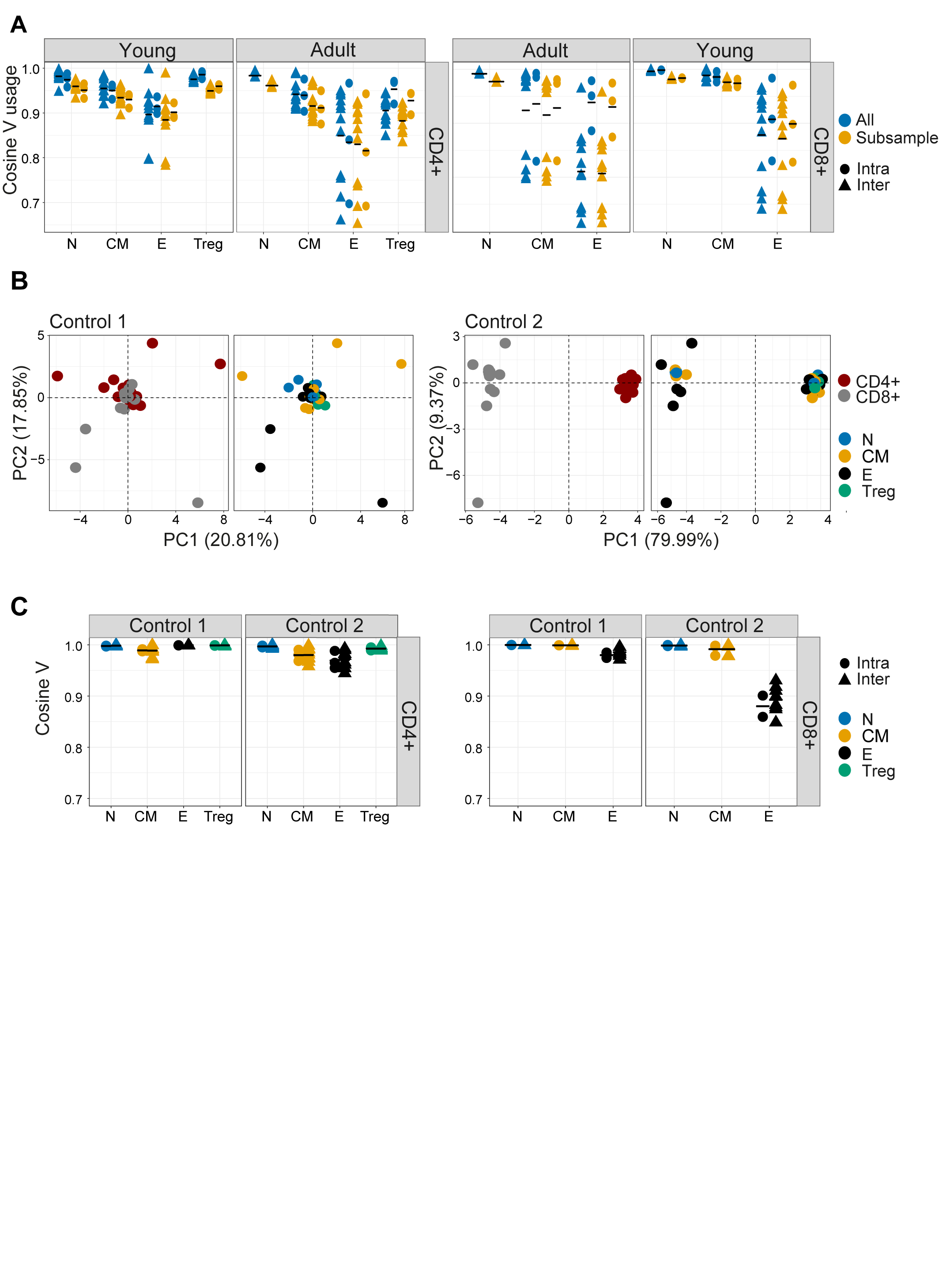

Supplement: Supplementary Figure 2.2 — (A) The impact of repertoire size on TCR V region similarity. TCR repertoires were subsampled as described in detail in Methods. The subsampling was repeated 100 times, and cosine similarity mean values in each metric were calculated. Pairwise cosine similarities of the TCRVβ usage calculated using all TCRs (blue) were plotted against pairwise cosine similarities of the TCRVβ usage calculated using equal numbers of subsampled TCRs (yellow). The inter-individual variability was calculated separately for spleen and bone-marrow. Each point is the cosine value calculated between two different mice and tissues (SP-SP, SP-BM, BM-BM). T cells compartments (colored dots) are divided into CD4+ (left) and CD8+ (right) from young or adult mice. Mean is shown by horizontal black lines. (B-C) Cosine similarities between TCRVβ frequency distributions in control repertoires. (B) PCA analysis of the TCRVβ usage distributions. Each color represents one compartment from one control repertoire (e.g., CD8+ Effectors, BM). See legend for symbols and color code. C) Pairwise cosine similarity scores between TCRVβ usage distribution in different control repertoires. Cosine scores between individuals (circles) or within individuals (between spleen and bone marrow, triangles). Each point is the cosine value calculated between two different repertoires (SP-SP, SP-BM, BM-BM). T cells compartments (colored dots) are divided to CD4+ (left) and CD8+ (right). Mean is shown by horizontal black lines. [file Image_5.tif]

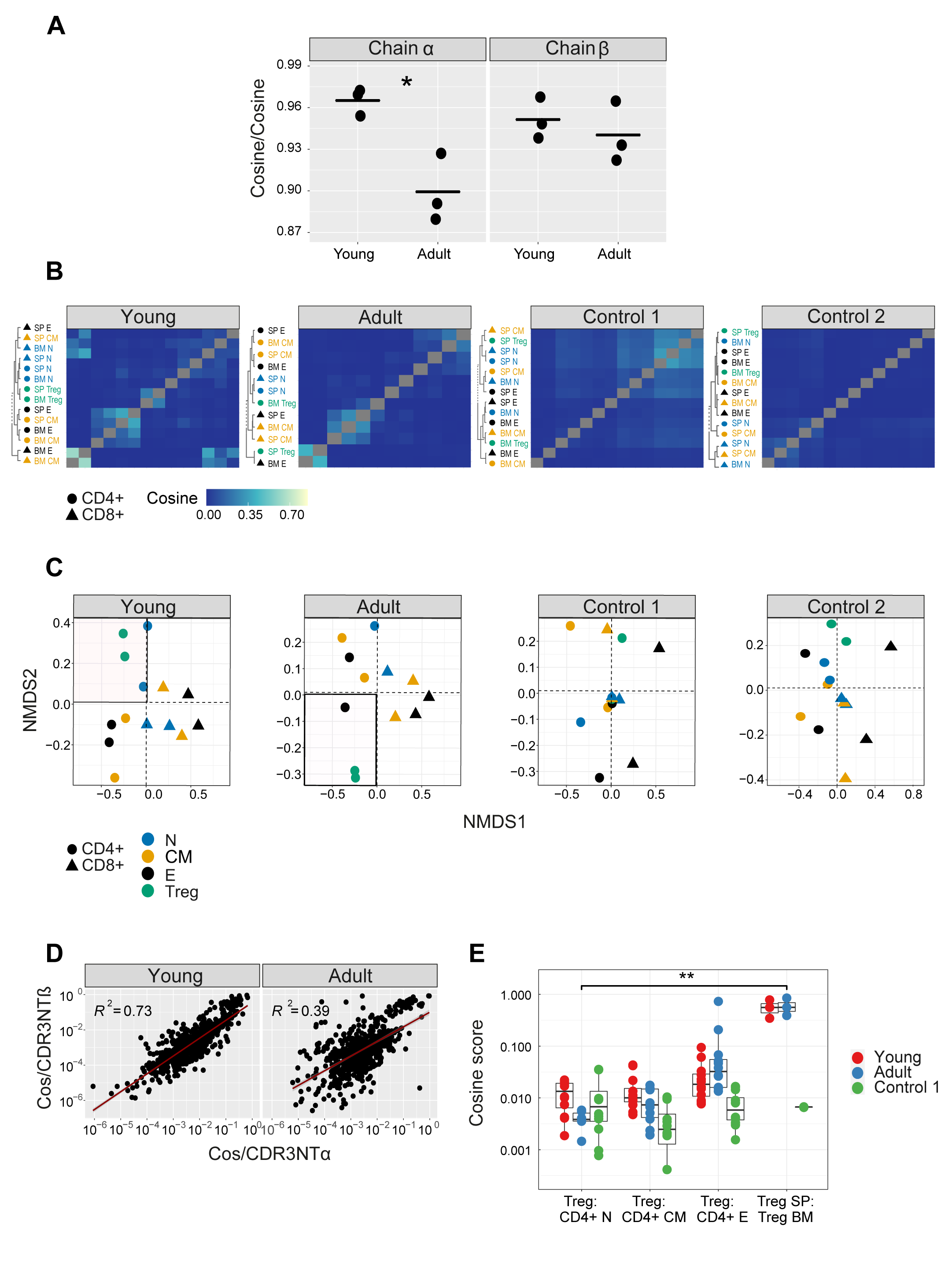

Supplement: Supplementary Figure 3.1 — Differential sharing of T cell CDR3 nucleotide α and β chain sequences in different subpopulations of T cells. (A) Cosine similarity for CDR3NT between all pairs of compartments within each young or adult mouse (for example, in young mouse 1: Treg SP and CD4+ N BM). These values were compared across mice using another Cosine score calculation. The color corresponds to the TCR chain (red= TCRα, grey= TCRβ). Significant differences between age groups are denoted in asterisks (P-values: *<0.05, **< 0.01, t-test). (B) Pairwise cosine similarity from representative young, adult, or control (“Control 1/2”) mouse CDR3αNT sequences. Correlation levels are represented by color (high=light blue, low= dark blue). In color and text, hierarchical clustering dendrograms for all T cell compartments are plotted to the left of each heat map (CD4+=circle, CD8+= triangles). (C) The similarity matrices shown as heatmaps in B are represented in two dimensions by NMDS. (D) CDR3αNT versus CDR3βNT cosine similarities between all pairwise compartments of young and adult mice. (E) Cosine index sharing levels between CDR3βNT of Tregs across tissues or naive and CD4+ effector repertoires within each young(red), adult(blue), or synthetic-based (green) mouse. Comparisons between the different tissues (SP-SP, SP-BM, BM-BM, n= 9). Mean is shown by horizontal black lines. Significant differences are denoted in asterisks (P-values: *<0.05, **< 0.01, t-test) and calculated between the groups: Tregs across tissues and Treg CD4+ naive cells. [file Image_6.tif]

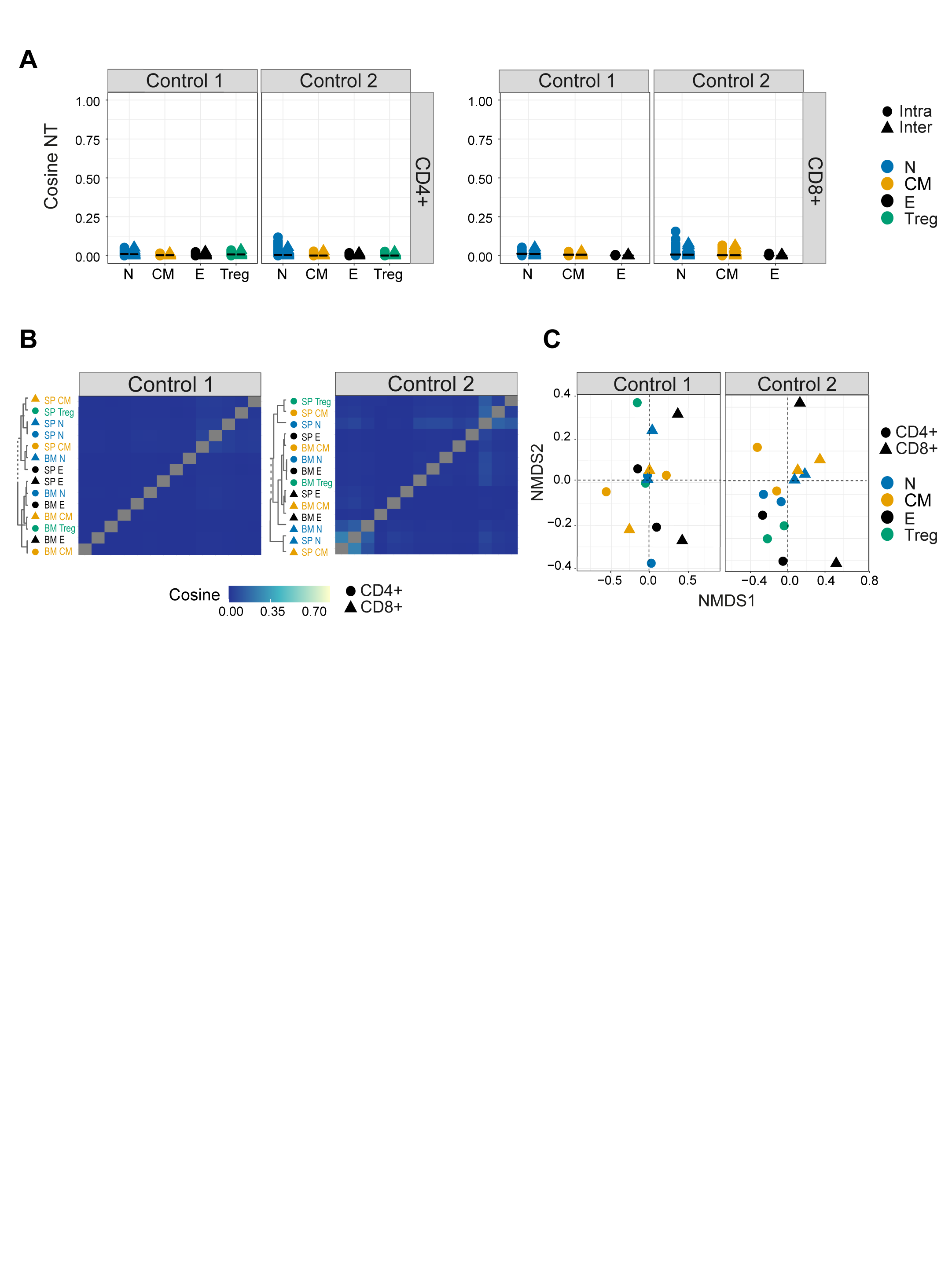

Supplement: Supplementary Figure 3.2 — Cosine similarities between CDR3βNT frequency distributions in control repertoires. (A) Pairwise cosine similarity scores between CDR3βNT distributions in different control repertoires. Cosine scores between individuals (circles) or within individuals (between spleen and bone marrow, triangles). Each point is the cosine value calculated between two different repertoires (SP-SP, SP-BM, BM-BM). T cells compartments (colored dots) are divided to CD4+ (left) and CD8+ (right). Mean is shown by horizontal black lines. (B) Pairwise cosine similarity from control 1 or 2 mouse CDR3βNT sequences. Correlation levels are represented by color (high=light blue, low= dark blue). In color and text, hierarchical clustering dendrograms for all T cell compartments are plotted to the left of each heat map (CD4+=circle, CD8+= triangles). (C) The similarity matrices shown as heatmaps in B are represented in two dimensions by NMDS. [file Image_7.tif]

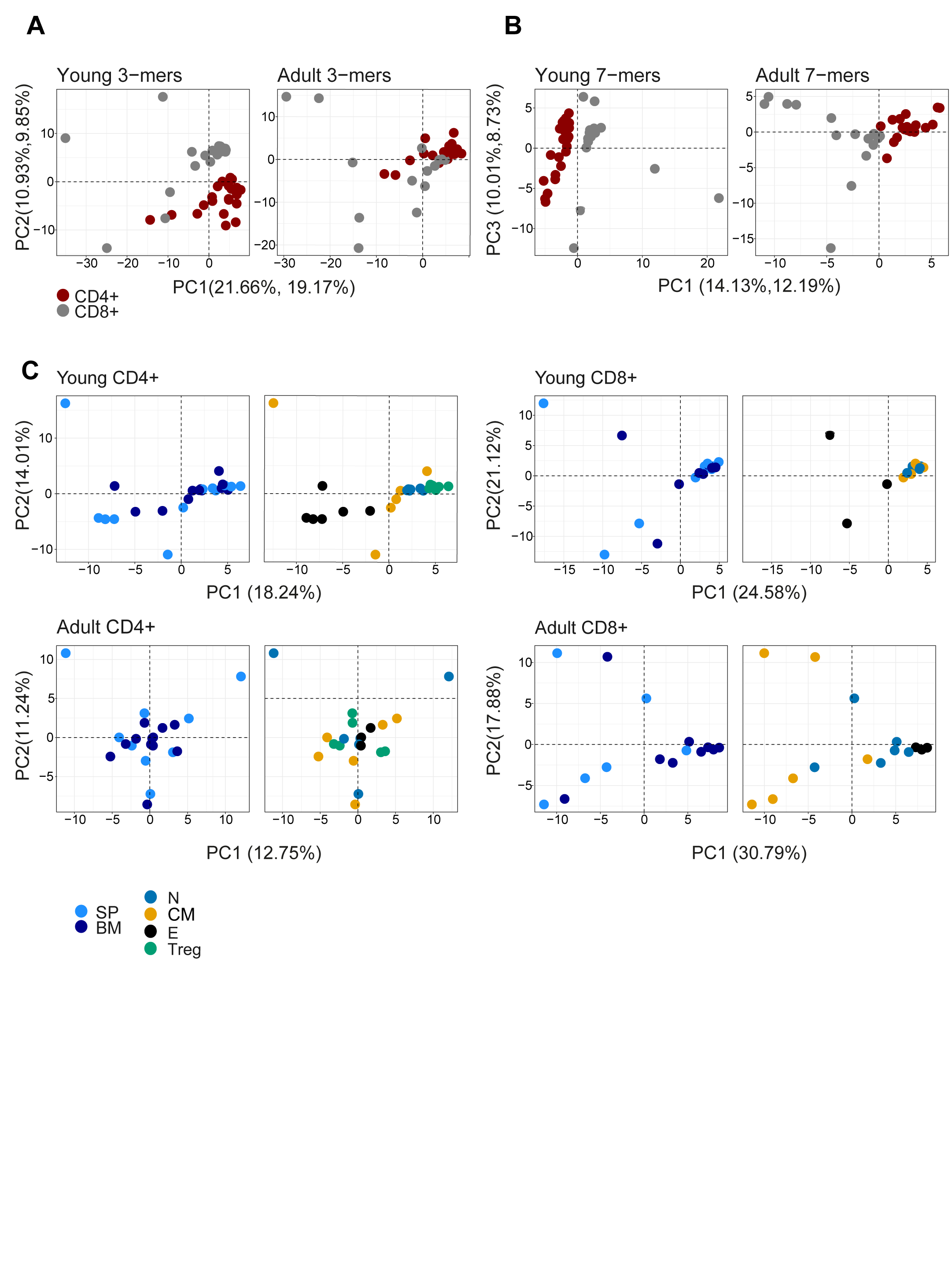

Supplement: Supplementary Figure 4.1 — PCA analysis of the frequency distributions of the most abundant CDR3βAA triplets and 7-mers. In panels A, B, the distributions are coloured according to CD4+ or CD8+ lineage. In panel C, CD4+ and CD8+ repertoires are plotted separately, and the individual points are coloured by tissue of origin or subpopulation. [file Image_8.tif]

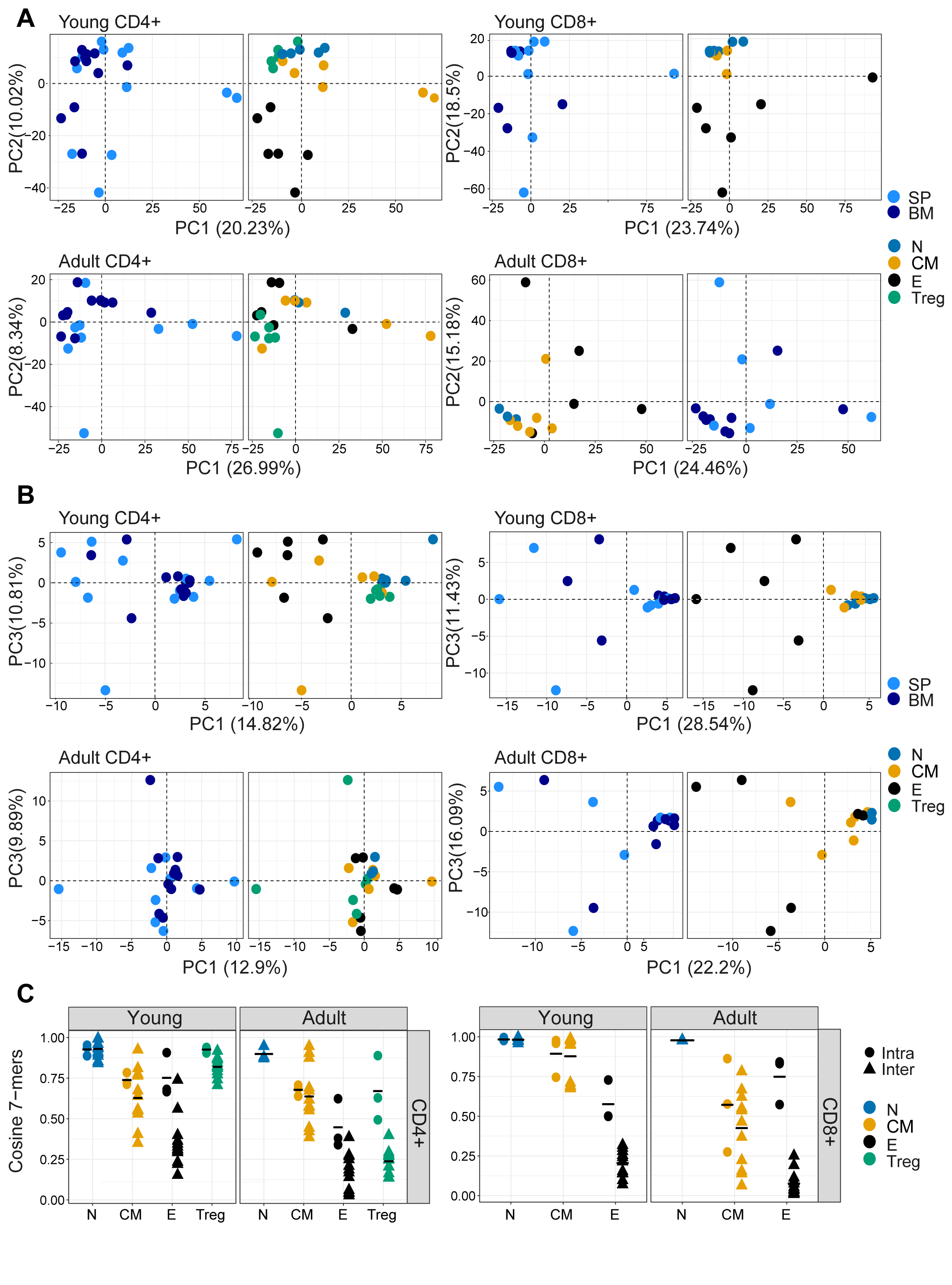

Supplement: Supplementary Figure 4.2 — (A)PCA analysis of the frequency distributions of the most abundant CDR3αAA triplets and 7-mers. (B)PCA analysis of the top CDR3αAA triplets. PCA analysis of the top 7-mers. CD4+ and CD8+ repertoires are plotted separately, and the individual points are coloured by tissue of origin or subpopulation. (C) Pairwise cosine similarities scores of the top 7-mers CDR3βAA motifs between individuals (circles) or within individuals (between spleen and bone marrow, triangles). T cells compartments (colored dots) are divided into CD4+ (left) and CD8+ (right) from young or adult mice. Mean is shown by horizontal grey lines. [file Image_9.tif]

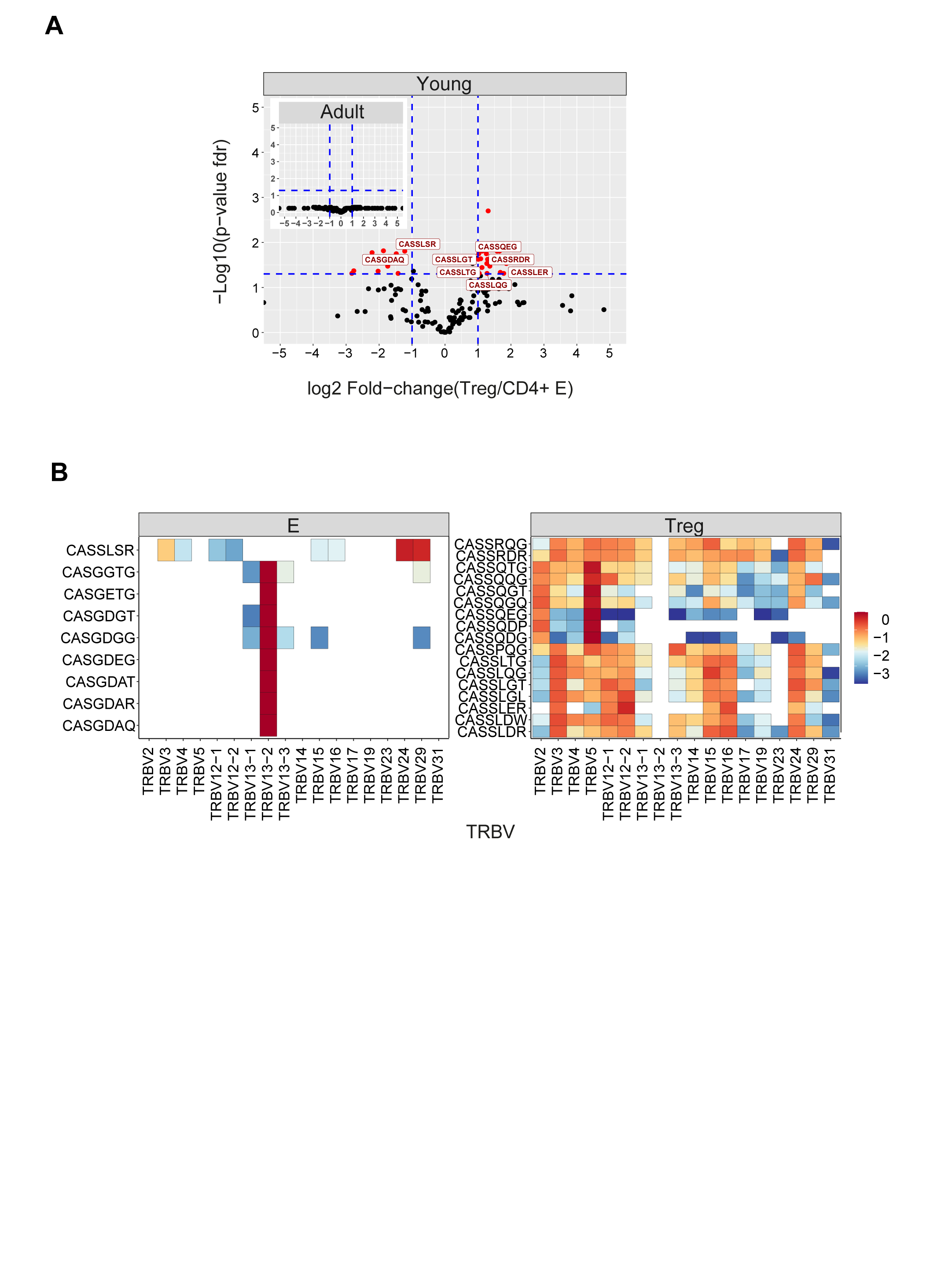

Supplement: Supplementary Figure 4.3 — Differential CDR3βAA 7mer frequency in CD4+ Treg and CD4+ Teff TCRs. (A) Each dot represents a single CDR3βAA 7-mer motif. P-value (t-test) was calculated for each motif across six samples (three mice and two tissues) of CD4+ Treg and CD4+ effector cells. The Y-axis shows FDR-adjusted p-values. The X-axis shows the log 2-fold-change, calculated between Treg and CD4+ effector mean motifs frequency across compartments (6 samples each). Significance thresholds are marked by the blue lines at y=1.3 (equivalent to a p-value of 0.05) and x= ± 1 (denoting a total fold-change of 2). Representative 7-mers above both thresholds are labeled with red text and dots. (B) The TCRVβ usage of the CD4+Treg (right) and CD4+ effector (left) differentially expressed 7-mers. The color represents the log10 frequency of each 7-mer in a specific Vβ gene (low= blue, high=red). [file Image_10.tif]

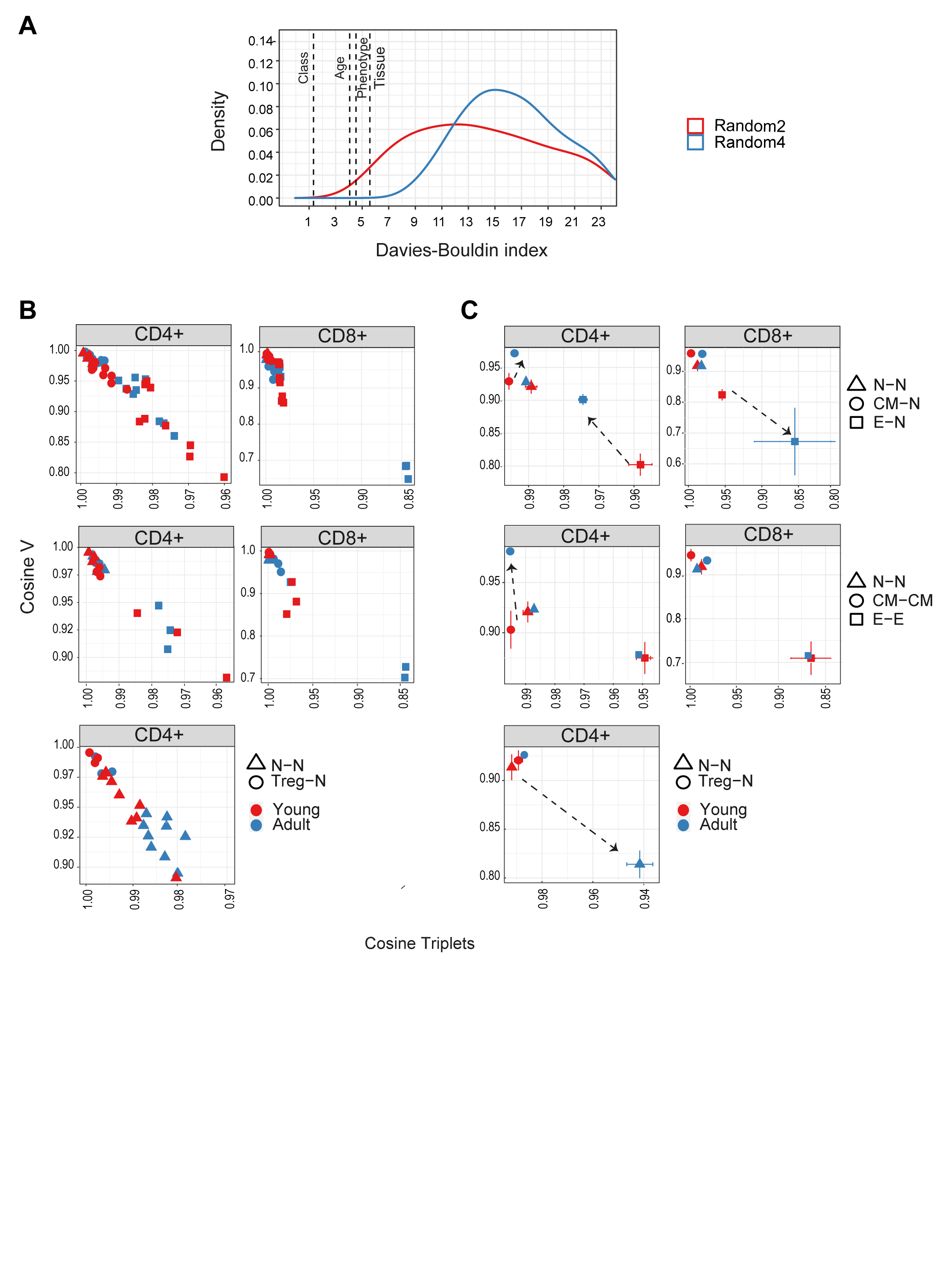

Supplement: Supplementary Figure 5.1 — (A) The Davies-Bouldin (DB) index applied to CDR3βAA top triplet motifs, capturing the average separability (ratio of the within-cluster variance to the separation between cluster centroids (lower score means better clustering) of clusters of different repertoires from their nearest counterpart. A reference distribution adding random clustering features (2 or 4 variables, red and blue lines respectively) to the same data and repeated the DB index calculation 10000 times. (B-C) V gene similarity plotted against CDRAA top triplet similarity distribution in young vs. adult or young. The β chain sequences of all pair cosine values in B. Mean cosine values for α chain sequences in C. Error bars are SEM. Comparing each repertoire to young naïve in CD4+, CD8+ and CD4+ Treg in young or adult mice (red dots = young mice, blue dots = adult mice). [file Image_11.tif]

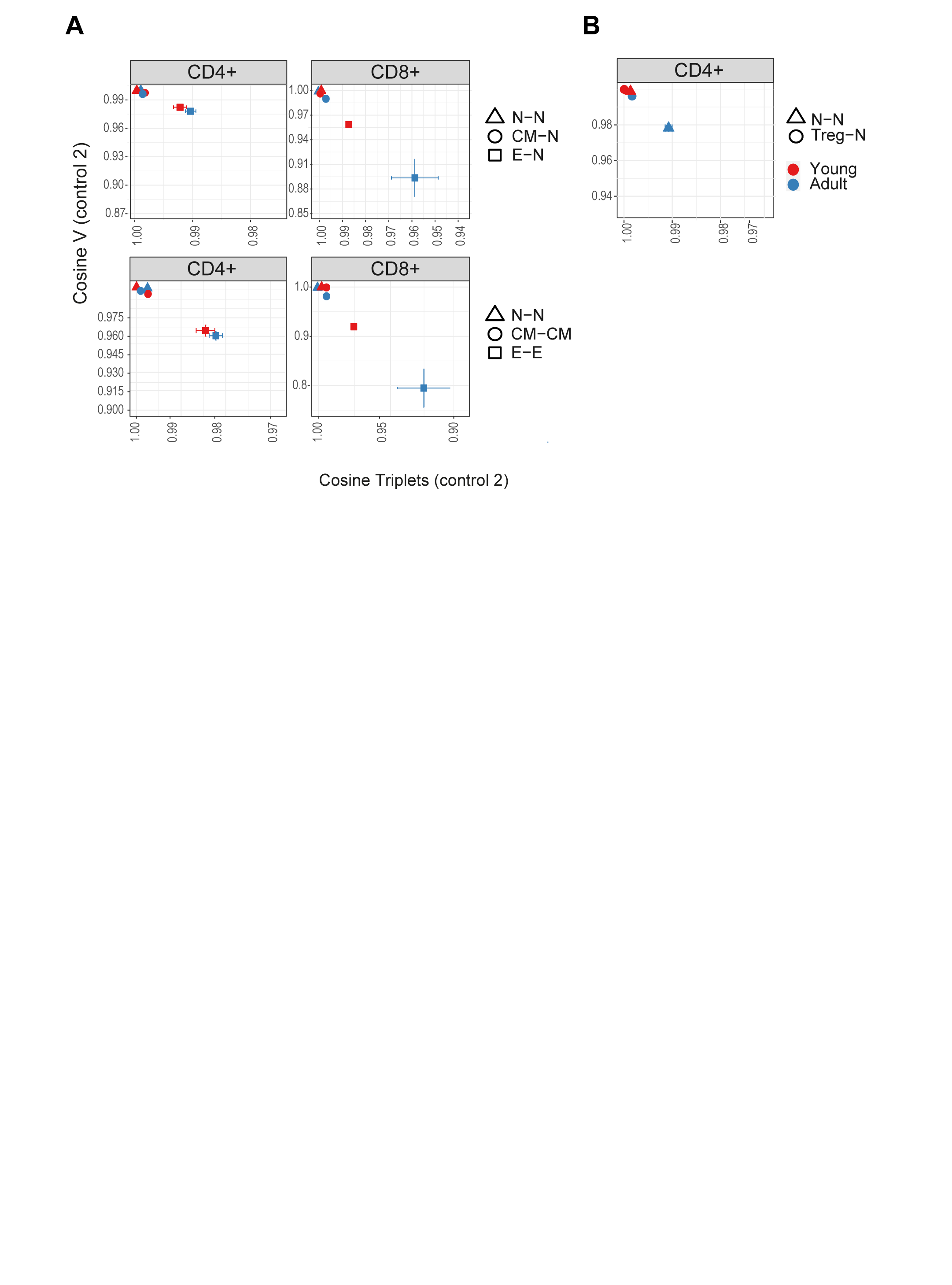

Supplement: Supplementary Figure 5.2 — TCRVβ gene similarity from control 2 repertoires plotted against CDR3βAA top triplet similarity distribution, comparing each repertoire to young naive in CD4+, CD8+ and CD4+ Treg in young and adult mice (red and blue dots). Each point is the mean cosine score, and the error bars are SEM. [file Image_12.tif]

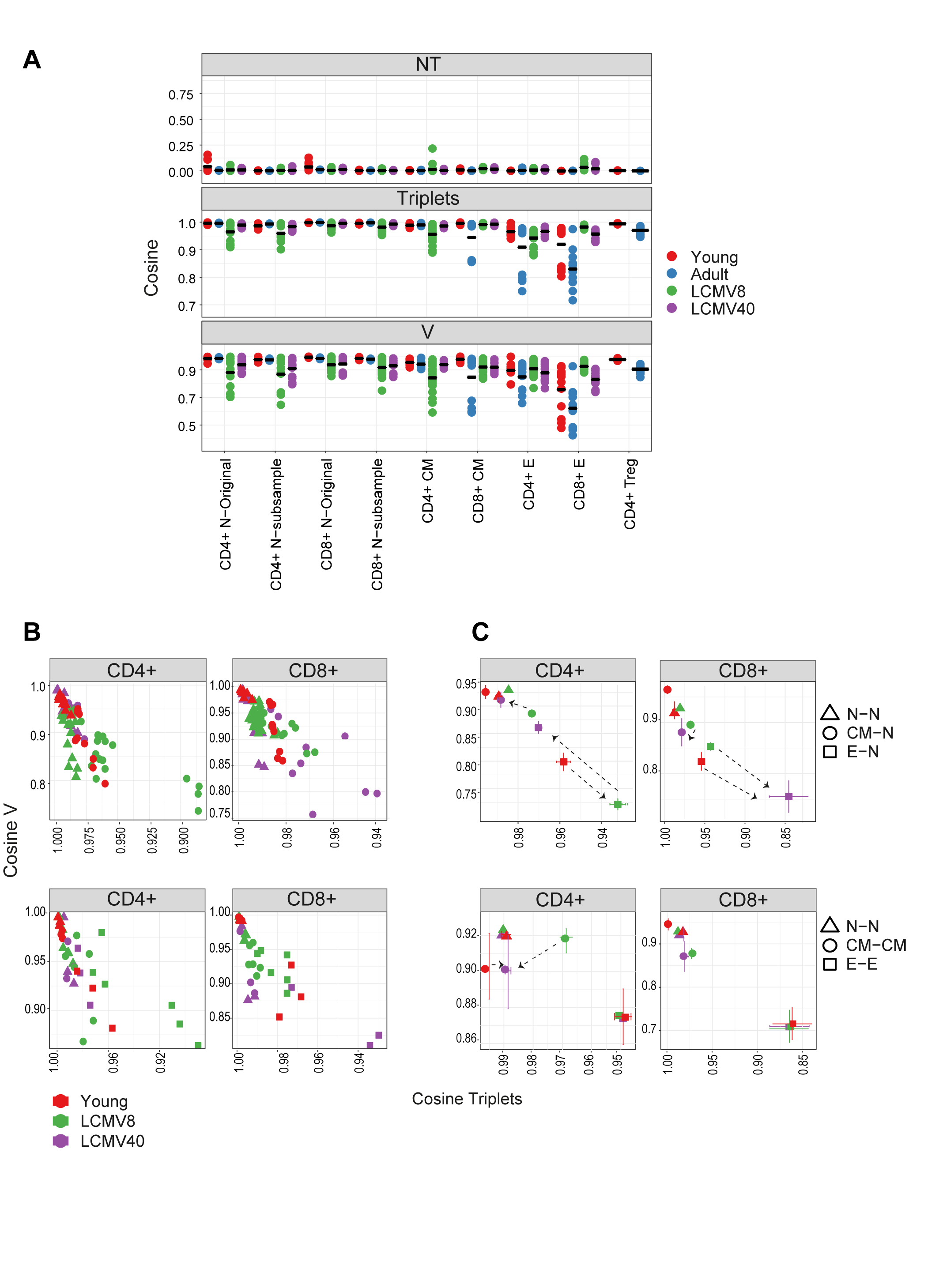

Supplement: Supplementary Figure 6.1 — (A) Cosine similarity index of TRBV genes, CDR3βNT, and top CDR3βAA 3-mers (top 350) motifs calculated between tissues and individuals. Colored dots reflect the mice groups (red = young, blue = adult, green/purple = mice after 8 and 40 days of acute LCMV infection, respectively). Horizontal black lines show the mean. The CD4+ and CD8+ naïve repertoires were subsampled as described in detail in Methods. The subsampling was repeated 100 times, and the mean values of each cosine similarity metric were calculated for TRBV genes, CDR3βNT, and top CDR3βAA triplets (350) motif distributions. (B-C) TCRV gene distribution similarity plotted against CDRAA top triplet similarity distribution in young vs. LCMV infected mice. The similarity between each repertoire and the young naïve in CD4+ and CD8+ repertoires to day 8 or 40 post LCMV infection (green or purple dots, respectively). The β chain sequences of all pair cosine values in B. Mean cosine values for α chain sequences in C. Error bars are SEM. [file Image_13.tif]

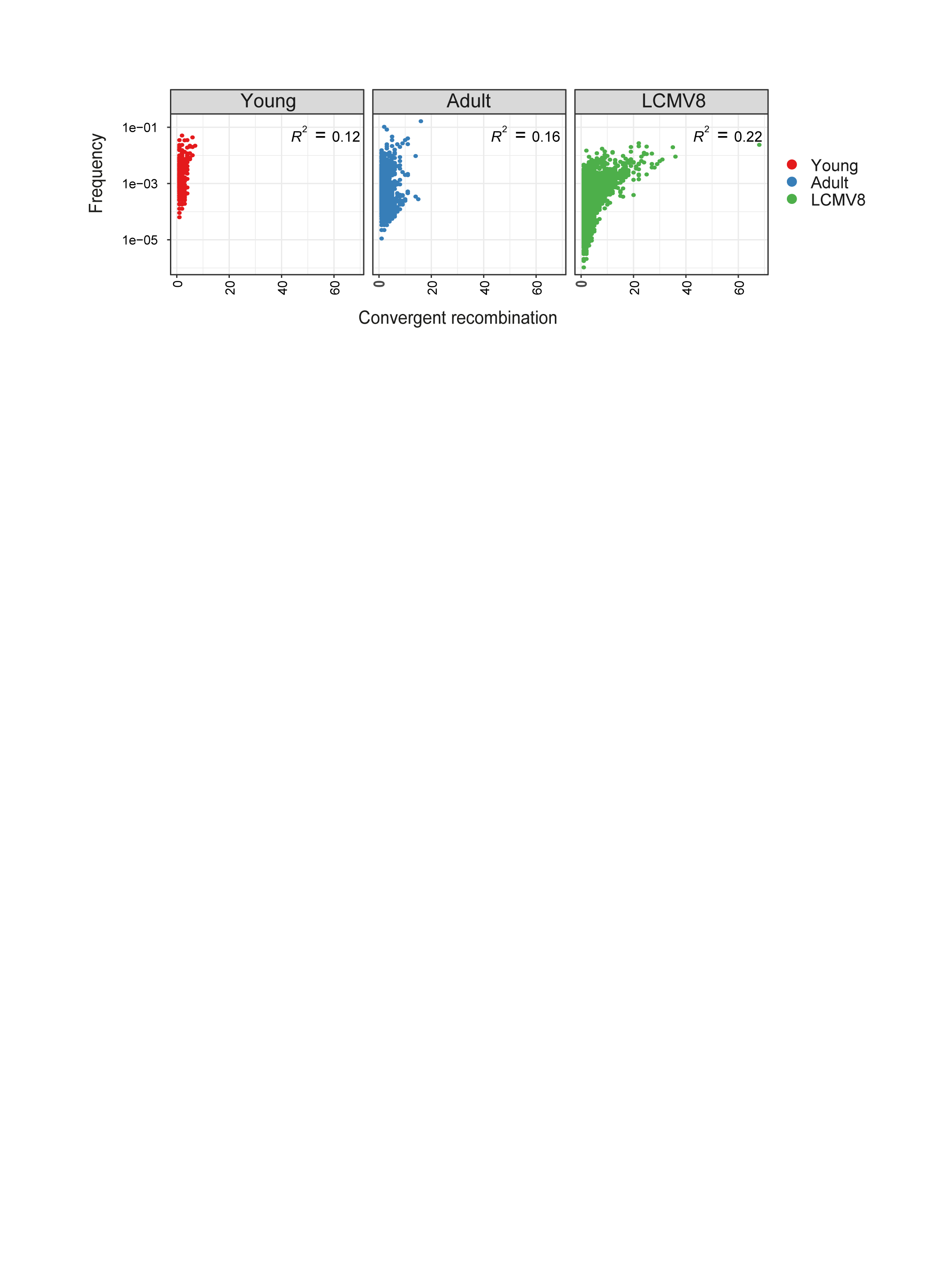

Supplement: Supplementary Figure 6.2 — Increased convergent recombination levels in CD8+ splenic effector cells with age and LCMV infection. Each point represents the number of CDR3NT_VJ which encodes for the same CDR3AAβ sequence (convergent recombination or coding-degeneracy) versus the frequency levels in young, adult, and mice after 8 days of LCMV infection (red or blue or green dots respectively). [file Image_14.tif]
